# Unusually Large Number of Mutations in Asexually Reproducing Clonal Planarian *Dugesia japonica*

**DOI:** 10.1371/journal.pone.0143525

**Published:** 2015-11-20

**Authors:** Osamu Nishimura, Kazutaka Hosoda, Eri Kawaguchi, Shigenobu Yazawa, Tetsutaro Hayashi, Takeshi Inoue, Yoshihiko Umesono, Kiyokazu Agata

**Affiliations:** 1 Global COE Program: Evolution and Biodiversity, Graduate School of Science, Kyoto University, Kitashirakawa-Oiwake, Sakyo-ku, Kyoto, Japan; 2 Department of Biophysics, Graduate School of Science, Kyoto University, Kitashirakawa-Oiwake, Sakyo-ku, Kyoto, Japan; 3 Cellular and Structural Physiology Institute, Nagoya University, Furo-cho, Chikusa-ku, Nagoya, Aichi, Japan; 4 Center for Developmental Biology, RIKEN, 2-2-3 Minatojima-Nakamachi, Chuo-ku, Kobe, Hyogo, Japan; 5 Advanced Center for Computing and Communication, RIKEN, 2–1 Hirosawa, Wako, Saitama, Japan; 6 Graduate School of Life Science, University of Hyogo, 3-2-1 Kouto, Kamigori-cho, Ako-gun, Hyogo, Japan; Institute of Crop Sciences, CHINA

## Abstract

We established a laboratory clonal strain of freshwater planarian (*Dugesia japonica*) that was derived from a single individual and that continued to undergo autotomous asexual reproduction for more than 20 years, and we performed large-scale genome sequencing and transcriptome analysis on it. Despite the fact that a completely clonal strain of the planarian was used, an unusually large number of mutations were detected. To enable quantitative genetic analysis of such a unique organism, we developed a new model called the Reference Gene Model, and used it to conduct large-scale transcriptome analysis. The results revealed large numbers of mutations not only outside but also inside gene-coding regions. Non-synonymous SNPs were detected in 74% of the genes for which valid ORFs were predicted. Interestingly, the high-mutation genes, such as metabolism- and defense-related genes, were correlated with genes that were previously identified as diverse genes among different planarian species. Although a large number of amino acid substitutions were apparently accumulated during asexual reproduction over this long period of time, the planarian maintained normal body-shape, behaviors, and physiological functions. The results of the present study reveal a unique aspect of asexual reproduction.

## Introduction

Planarians are non-parasitic flatworms found throughout the world, and include species that inhabit freshwater, seawater, and wetland. Freshwater planarians are most common, with more than 200 species identified to date [1]. They are generally highly regenerative [2–5]. This outstanding regeneration capability is made possible by adult pluripotent stem cells, called neoblasts, that account for approximately 30% of the total cells [6]. Also, despite their rather simple body structure and behaviors, planarians have well-organized brains [7, 8]. Some planarian species can switch between asexual and sexual reproduction depending on the season and on circumstances such as food conditions and temperature [9]. When the temperature rises in the summer, the reproductive organs degenerate, and the planarians then undergo asexual reproduction by fission, resulting in an increased number of individuals [10, 11]. In addition, some strains/colonies propagate solely through asexual reproduction without undergoing sexual/asexual cycles [12, 13]. Several planarian species, such as *Dugesia japonica* and *Schmidtea mediterranea*, can easily be bred under laboratory conditions by asexual reproduction [14, 15]. *D*. *japonica* inhabits East Asia, and is the most commonly found species in Japan [16]. Its body is around 8–25 mm long, with two eyes in its triangle-shaped head, and it has been used in many studies on development and regeneration. Recently, planarians have become attractive experimental animals not only for regeneration biologists but also for neurobiologists, and the number of researchers utilizing planarians is rapidly increasing. However, the available planarian genome assembly remains very crude [17], which presents a barrier to precise molecular analyses. It is very difficult to assemble planarians' genomes due to the presence of large numbers of repetitive sequences, although many researchers have extensively sequenced their genomes using next generation sequencing (NGS). We must overcome these problems to provide useful databases for planarian researchers.

We previously reported a comparative analysis of homologous genes between two planarian species that showed there were many between-species differences, including amino-acid substitutions, in genes involved in metabolic and defense systems, which we speculated were a consequence of adaptation to different living conditions [18]. However, how long-term asexual reproduction affects planarian genes remains to be elucidated.

To clarify how asexual reproduction affects planarian genes over a long period, an asexually reproducing strain was established from a single individual of *D*. *japonica*, and maintained in the asexual state under fixed breeding conditions for more than 20 years. Since the genome sequence of *D*. *japonica* has not been determined yet, we first conducted large-scale genome sequencing by NGS and attempted *de novo* assembly. High-throughput NGS enables not only the determination of a previously unknown genome sequence, but also comprehensive transcriptome analysis covering low-expression genes. We developed a new algorithm to construct what we call a “Reference Gene Model”, and investigated the mutations in detail through gene-level quantitative mutation analysis of numerous genes.

## Results

### Genome Size Estimation for *D*. *japonica*

**Fig 1:**
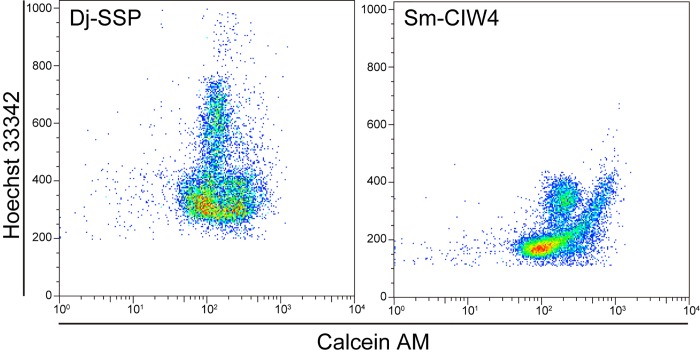
FACS profiles of cells derived from *D*. *japonica* and *S*. *mediterranea*. Fluorescence-activated cell sorting (FACS) profiles of cells derived from the whole body of *D*. *japonica* SSP-strain (Dj-SSP) and *S*. *mediterranea* CIW4-strain (Sm-CIW4). The number of cells analyzed was 9,104 and 11,588, respectively. Each dot indicates the relative fluorescence intensity of Calcein AM and Hoechst 33342, and red color indicates a relatively high population of cells. Calcein AM labels the cytosol of viable cells, and its intensity is plotted on a logarithmic scale on the X-axis. Hoechst 33342 labels chromosomes, and is used to estimate the genome size, and its intensity is plotted on a linear scale on the Y-axis.

While the karyotype of *D*. *japonica* has been reported to be 2n = 16 [19], its genome size has not been determined. In *S*. *mediterranea*, another planarian species, the number of chromosomes is 2n = 8, and the genome size has been estimated to be around 480 Mb [17]. To estimate the genome size before whole genome sequencing of *D*. *japonica*, cells isolated from adults of *D*. *japonica* SSP-strain (Dj-SSP) and *S*. *mediterranea* CIW4-strain (Sm-CIW4) were subjected to double-staining with Hoechst 33342 and Calcein-AM followed by FACS (fluorescence activated cell sorting) analysis. Dj-SSP and Sm-CIW4 are clonal strains derived from single individuals by maintaining asexual reproduction throughout all the generations cultured in the laboratory thus far (see Materials and Methods). The FACS analysis showed that the Hoechst fluorescence distribution in *D*. *japonica* was shifted to indicate approximately 1.9-fold greater intensity compared with that in *S*. *mediterranea* (Fig 1). Since the Hoechst fluorescence intensity roughly correlates with the genome size, these results suggested that the genome size of *D*. *japonica* was about 900 Mb.

### Genome Sequencing and Quality Control

Genomic DNA was extracted from Dj-SSP adults to prepare three genomic DNA libraries differing in fragment size (300, 350 and 400 bp). For each library, 150 bp paired-end sequencing in a total of 7 lanes was carried out with an Illumina GAIIx DNA sequencer, and an 89.3 Gbp raw DNA sequence with a total of 595.2 million reads was obtained (S1 Table). Given the genome size estimated by FACS analysis, this number of bases corresponds to a mean coverage of 99X. Next, to verify the optimal data set for *de novo* assembly, the raw data acquired were subjected to the following three quality control procedures to obtain the respective valid sequences. Quality-value-based trimming of the sequences yielded 585.9 million reads in total, and valid reads of a total of 74.3 Gbp. Using a technique of overlapping and merging the sequences of the same DNA clone in each genomic library, 111.6, 71.1, and 43.7 million merged reads were obtained with insert sizes of 300, 350, and 400 bp, respectively, making the total number of bases 44.8 Gbp. The number of read pairs that were not merged was, respectively, 14.7, 17.2, and 38.8 million, and the total number of bases that were not merged was 21.2 Gbp. Error correction based on the frequency information of the k-mer gave 73.0 Gbp of valid data, with a total of 575.9 million reads.

### K-Mer Optimization and Characterization of the Planarian Genome

**Fig 2:**
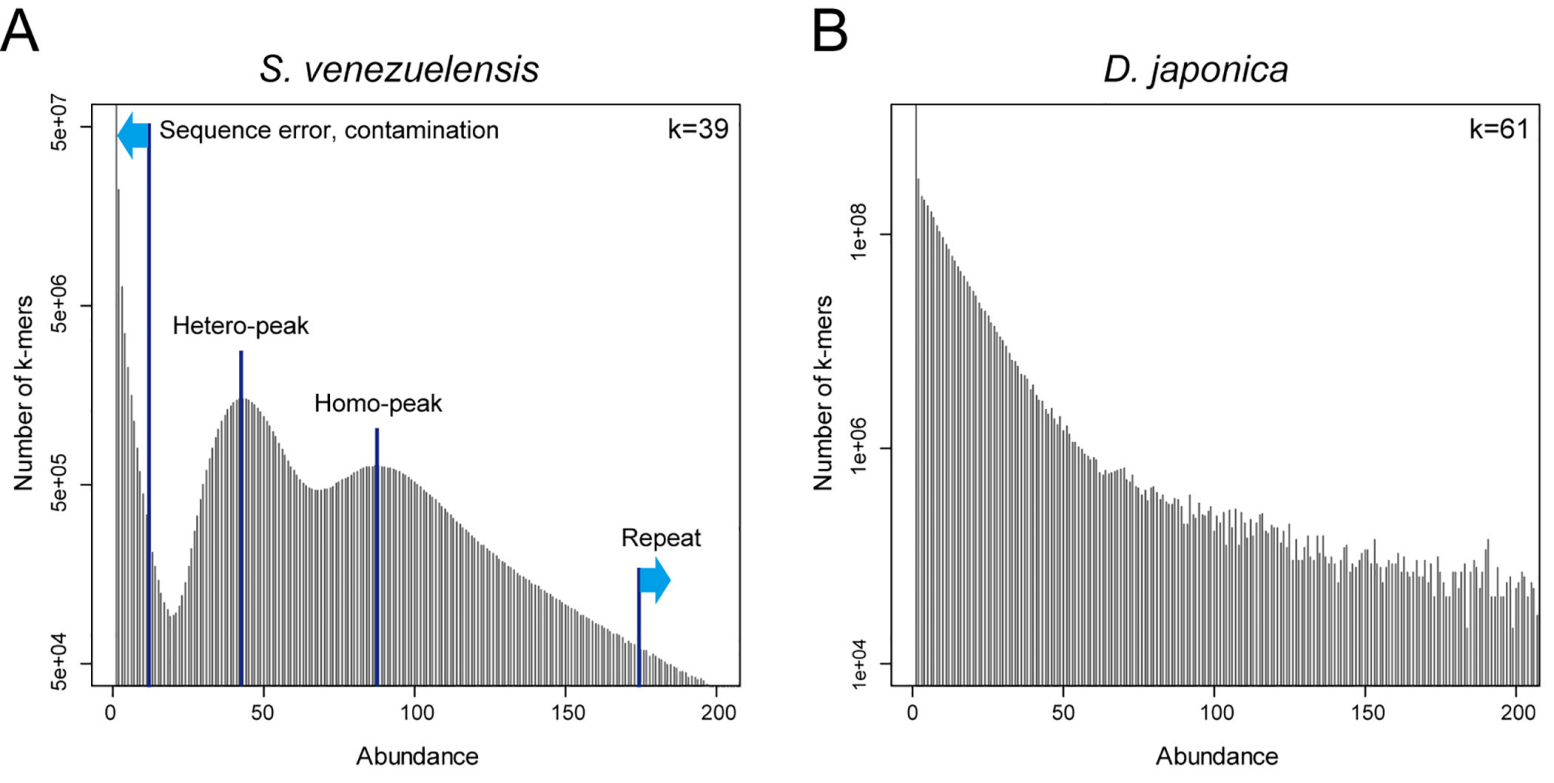
k-mer spectra of *D*. *japonica* and control genome. (A) shows the control results for *S*. *venezuelensis*, which is known to be highly heterozygous (0.927%), and showed a bimodal peak consistent with its genome characteristics. (B) shows the results for *D*. *japonica*, and neither a monomodal nor a bimodal peak was found.

Prior to the *de novo* genome assembly, abundance histograms were constructed with k-mer values ranging from 21 to 121, to optimize the k-mer value for the de Bruijn Graph algorithm [20] to be used in the assembly program, and to estimate the complexity of the *D*. *japonica* genome. Fig 2A shows the results of the genome data analysis for *Strongyloides venezuelensis* (an infectious nematode), which is known to have a highly heterozygous genome (0.927%) and was used as a control. Fig 2B shows the results for the *D*. *japonica* genome obtained using the sequence from the quality value-based trimming. Normally in k-mer histogram analysis, a monomodal (low heterozygosity) or bimodal (high heterozygosity) peak is observed at a specific abundance depending on genome size and input sequence quantity, after the noise data (low-frequency contamination or sequence errors) are observed [21, 22]. While *S*. *venezuelensis* showed a bimodal peak consistent with its genome characteristics, neither a monomodal nor a bimodal peak was found for the planarian, and there was no noticeable boundary between the signal and the noise. Moreover, the high-abundance fraction remained at a high level, which indicates that the genome contains a significant number of high-frequency repeats. Although the analysis was conducted using a wide range of k-mer values, all the values gave similar results (S1 Fig).

### *De Novo* Genome Assembly

For *de novo* genome assembly, we used two assembly programs, SOAPdenovo [23] and Platanus [22]. Platanus is known to be a powerful assembly software for highly heterozygous genomes. The sequence data used were the three QC data sets described above. Since SOAPdenovo requires a fixed k-mer value, we used the optimum value estimated with the KmerGenie program [21]. S2 Table shows the assembly results obtained with the two programs. Only very short contigs/scaffolds were obtained regardless of the assembler used, and Platanus did not produce valid results except in the case of the Error Correction data set. Performance was not improved at all even by using the Error Correction data set corrected with an assumption that k-mer values occurring at low frequency represent sequencing errors, or by adding a higher heterozygous option to the Platanus program.

### Transcriptome Sequencing and Assembly

Next, we attempted a large-scale transcriptome analysis to investigate whether the sequence diversity suggested by the results of k-mer analysis and genome assembly was present in gene regions or not. Additionally, in order to compare the results of our previous EST (expressed sequence tag) analysis, the same *D*. *japonica* GI-strain (Dj-GI) that had been used for the EST analysis was used for the present transcriptome analysis. RNA was extracted from pieces of head (HP), and also from the anterior blastema (AB) and posterior blastema (PB) that had formed in regenerated planarians at 24 hours after amputation (see Materials and Methods). cDNA libraries for Illumina MiSeq and Roche 454 were then constructed using these extracted RNA preparations. With MiSeq, a total of 3 runs (1 run for each library) of 251-bp paired-end sequencing were performed (Table 1). For HP, AB, and PB, 16.1, 15.4, and 13.0 million read pairs were obtained, respectively, and the data of 16.5 Gbp were acquired in total. After quality and adapter trimming, valid data of 87.8 million reads and 15.3 Gbp in total were obtained, with mean base length 175 bp. With 454, a total of 5 runs of single-end sequencing were performed for HP (1.5 runs), AB (1.75 runs), and PB (1.75 runs), to obtain 2.0, 2.2, and 2.1 million reads, respectively (Table 1). The total number of bases was 4.4 Gbp. After removing low quality parts and adapter sequences, valid data of 6.3 million reads and 3.2 Gbp in total were obtained, with mean base length 507 bp.

**Table 1:** Information about transcriptome sequences.

Sequencer	Tissue	Mean length in library (bp)	# of runs	Read Type	# of raw reads	Total raw reads (bp)	# of trimmed reads	Mean of trimmed reads (bp)	Total trimmed reads (bp)
MiSeq	Head piece (HP)	339	1	Forward	16087607	2973194757	15862442	176	2798435158
				Reverse	16087607	2983298017	15862442	174	2757739084
	Anterior blastema (AB)	338	1	Forward	15392786	2882472185	15185359	182	2758172139
				Reverse	15392786	2945476397	15185359	169	2559496061
	Posterior blastema (PB)	316	1	Forward	12963014	2358665075	12840281	175	2252426852
				Reverse	12963014	2366665689	12840281	171	2200488112
	Total		3		88886814	16509772120	87776164	175	15326757406
454	Head piece (HP)	1002	1.5	Forward	2019370	1421627103	2019295	528	1066140982
	Anterior blastema (AB)	984	1.75	Forward	2162308	1461483552	2161986	493	1066106705
	Posterior blastema (PB)	967	1.75	Forward	2145206	1490400204	2144960	500	1073521446
	Total		5		6326884	4373510859	6326241	507	3205769133

First, the Trinity program [24] was used for the transcriptome assembly of MiSeq reads. Because sequence errors not caught with the quality value-based assessment may present in later cycles of sequencing (3' end), we also conducted assembly of the sequence trimmed to 200 bp of the initial sequencing cycles (5' end). The results from the assembly had a mean isotig length of 626 bp, which corresponds to an mRNA isoform unit and was shorter than the reported mean length of 941 bp for the EST assembly [18]. The isogroup number was 144,841, which corresponds to gene units and was markedly larger than the expected number of genes (Table 2). Using the data with sequence quality enhanced by trimming to the 5'-end 200 bp did not improve the isogroup number or the isotig N50 value. These results can be explained by the fact that the gene sequence, which was originally a single unit, was divided into multiple sub-sequences, and consequently the length was shortened and the total isogroup number was increased.

**Table 2:** Statistics of transcriptome assembly.

Data set	Assembler	# of isogroups (gene)	# of isotigs (variant)	Isotig N50 (bp)	Mean isotig length (bp)	Total isotig length (bp)
MiSeq	Trinity	144841	199209	975	626	124864539
MiSeq trim 200 bp	Trinity	137505	190374	1034	643	122489178
MiSeq trim 200 bp + 454	Trinity	139428	208851	958	660	137923333
454	Newbler	27910	58202	2496	1891	110078367

Due to the difference of the terms of the assembled sequence between Trinity and Newbler, "isogroup" is defined as "gene-group", and "isotig" is defined as "gene-isoform".

Second, we conducted assembly of the 454 sequences with Newbler [25]. The results showed mean isotig (gene isoform) length of 1,891 bp, and isogroup (gene) number of 27,910 (Table 2). These values were both superior to those from the MiSeq assembly, and the total base number of isotigs did not appreciably differ from that obtained with MiSeq. Thus, high-quality data with fewer disruptions of the gene sequence were obtained with Newbler. Hybrid assembly of 454 and MiSeq was attempted with Trinity, but no useful results were obtained (Table 2).

### The Reference Gene Model

**Fig 3:**
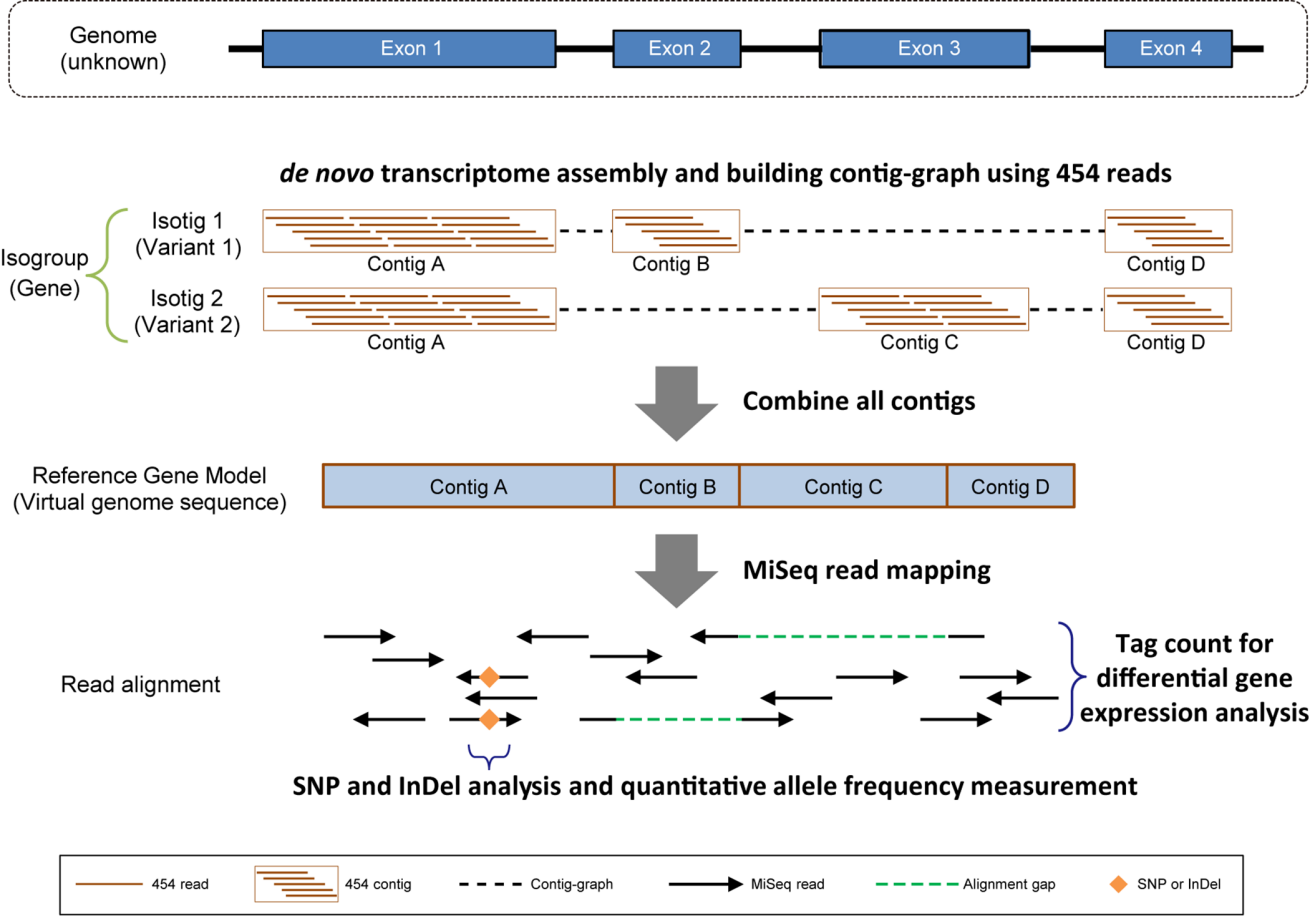
Schematic overview of the Reference Gene Model. Alternative splicing produces multiple transcript variants of mRNA isoforms from a single gene locus. Because the transcriptome sequences are derived from each isoform, the reads of a common exon map to multiple variants of the reference during the mapping process. To solve this multi-mapping problem, the assembly contigs are combined according to the information of the contig-graph that is constructed by the Newbler program in the process of *de novo* assembly. The combined contigs, which are called the Reference Gene Model, display the virtual genome sequence without introns. By mapping the MiSeq RNA-seq reads to this model, it becomes possible to count reads, detect SNPs (single nucleotide polymorphisms), and analyze mutations quantitatively in the unit of a gene. Variant-derived sequences, which do not have specific exons, are mapped to the model using a local alignment algorithm to overcome exon gaps.

In general, transcriptome analysis by NGS uses a known genome sequence and information of gene regions as references, and maps short sequence reads to the references to perform gene expression analysis [26, 27]. However, the genome assembly results obtained in the present study were not sufficient to use as references. We therefore tried to use the transcriptome assembly as the reference for genes. For this attempt, a different algorithm was required because of the multiple splice variants transcribed from a single gene locus on the genome, which causes multi-mapping reads in exons shared among isoforms when RNA reads are simply mapped to the transcriptome assembly. Therefore, we devised the "Reference Gene Model", a virtual genome sequence set without introns, by linking exons linearly in the order found in the genome based on the contig-graph information obtained in the course of 454 sequence assembly (Fig 3). Because the Reference Gene Model is a virtual genome sequence, all isotigs constituting a single gene model were subjected to homology search against the NCBI NR database, and the isotig with the highest score was chosen as a representative sequence of the gene. In homology search of 27,910 isogroups using BLASTX [28], 13,796 (49.4%), 17,212 (61.7%), and 16,950 (60.7%) isogroups were hit with an E-value of 1e-5 or less against the SWISS-PROT, UniProt TrEMBL, and NCBI NR databases, respectively.

### Verification of Accuracy of the Reference Gene Model by Differential Gene Expression Analysis

To verify the accuracy of the Reference Gene Model, we attempted quantitative differential expression analysis. For expression level analysis, we used the MiSeq data, which had superior read depth. The mapping rates of HP, AB, and PB reads were 92.5%, 91.2%, and 88.6%, respectively (S2A Fig). Then, genes expressed at different levels in AB and PB were identified. As a result of normalization and testing with the DEGseq program [29], 3,420 genes were considered to be genes with a difference in expression level between the two libraries (S2B and S2C Fig). After filtering and gene annotation, finally 117 genes were extracted as genes with significantly differential expression in the anterior blastema (S3 Table). These genes included a large number of genes which have been shown to be expressed specifically in the head: *six3-1*, *runt-1*, *rho*, *E3 ubiquitin-protein ligase* and *Solute carrier family 43* [30–34]. Especially notably, the list contained a number of genes which are thought to be involved in the formation of the brain rudiment during regeneration, including *nou-darake*, *Dj*z*icA*/*B*, *tlx-1*, *DjwntB*, *DjfzA* and *DjsFRP-A* [35–38].

Seventeen genes with a large expression difference were chosen, and their expression levels were examined by qRT-PCR (quantitative real-time PCR) to confirm the significance of these data. The results revealed that many genes showed a correlation between RNA-seq- and qRT-PCR-based quantification results (S2D Fig).

### SNP Calling

**Fig 4:**
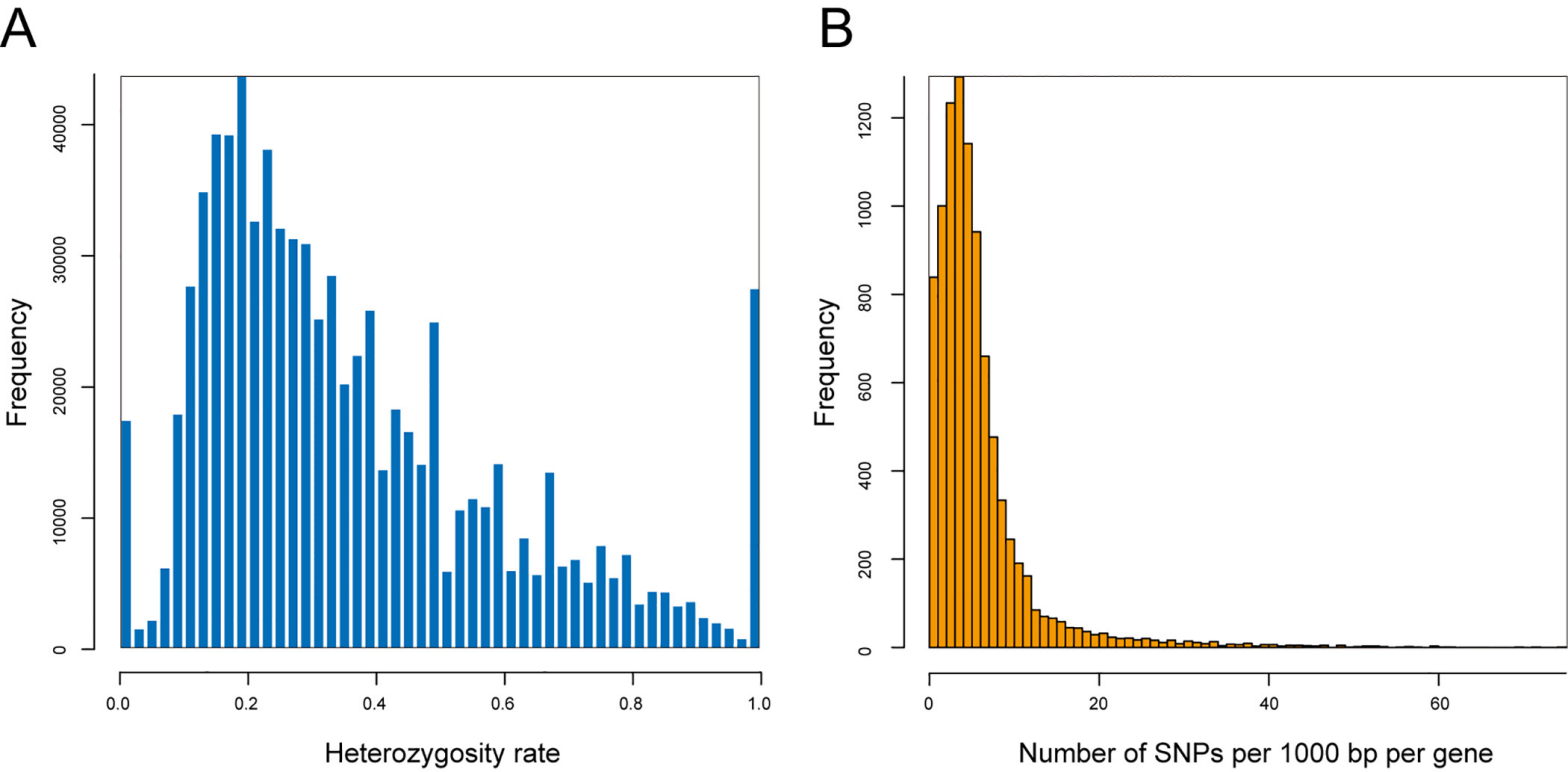
Summary of SNP analysis. (A) Histogram of the heterozygosity rate for SNPs. Typically, SNPs with variant ratio close to 1.0 are considered homozygous SNPs, and SNPs with a narrow normal distribution that peaks at 0.5 are considered heterozygous SNPs. (B) Histogram of SNP number per gene per 1000 bp.

The results from the genome and transcriptome analyses suggested the possibility that a large number of mutations were distributed not only outside the gene regions but also within coding regions. To investigate SNPs in individual genes in detail, the MiSeq transcriptome sequences of HP, AB, and PB were mapped to the Reference Gene Model, and SNP calling was performed with GATK [39]. As a result, a large number of SNPs (791,857) were detected, despite the fact that the samples were from completely clonal populations. Moreover, mutations were found in 26,060 genes, which account for 93.4% of all genes analyzed, and were mostly heterozygous SNPs (Table 3). Fig 4A shows a histogram of the heterozygosity rate of mutations relative to the references. In general in SNP analysis, SNPs with a reference–sample heterozygosity rate close to 1.0 are considered to be homozygous SNPs, while those represented by a narrow normal distribution that peaks at 0.5 are considered to be heterozygous SNPs. The present result, however, was markedly different. The references used in the analysis comprised the 454 sequencing results from sequencing of RNA sources identical to those used for MiSeq. Therefore, SNPs with a heterozygosity rate close to 1.0 had a low read coverage in the reference side (454) and could not represent major alleles, or were the result of specific biases between sequence platforms, rather than biological genotypes. Furthermore, we could not rule out the possibility that SNPs with a ratio close to 0.5 include heterozygosities that the ancestral individual originally possessed. After removing them, 400,618 SNPs remained, and the number of mutated genes became 23,925 (85.7%) (Table 3). This number of SNPs corresponds to 9.21 SNPs / 1000 bp.

**Table 3:** Summary of SNP analysis.

**Zygosity type**	**# of SNPs**		**# of SNPs filtered**	
All SNPs	791857			
Homozygous SNPs	33663	4.3%		
Heterozygous SNPs	758194	95.7%	400618	50.6%
**Zygosity type**	**# of genes**		**# of genes filtered**	
All genes	27940			
SNP-containing genes	26060	93.4%		
Heterozygous SNP-containing genes	25792	92.4%	23925	85.7%

### SNP Frequency and Commonality among Sequencing Platforms

**Fig 5:**
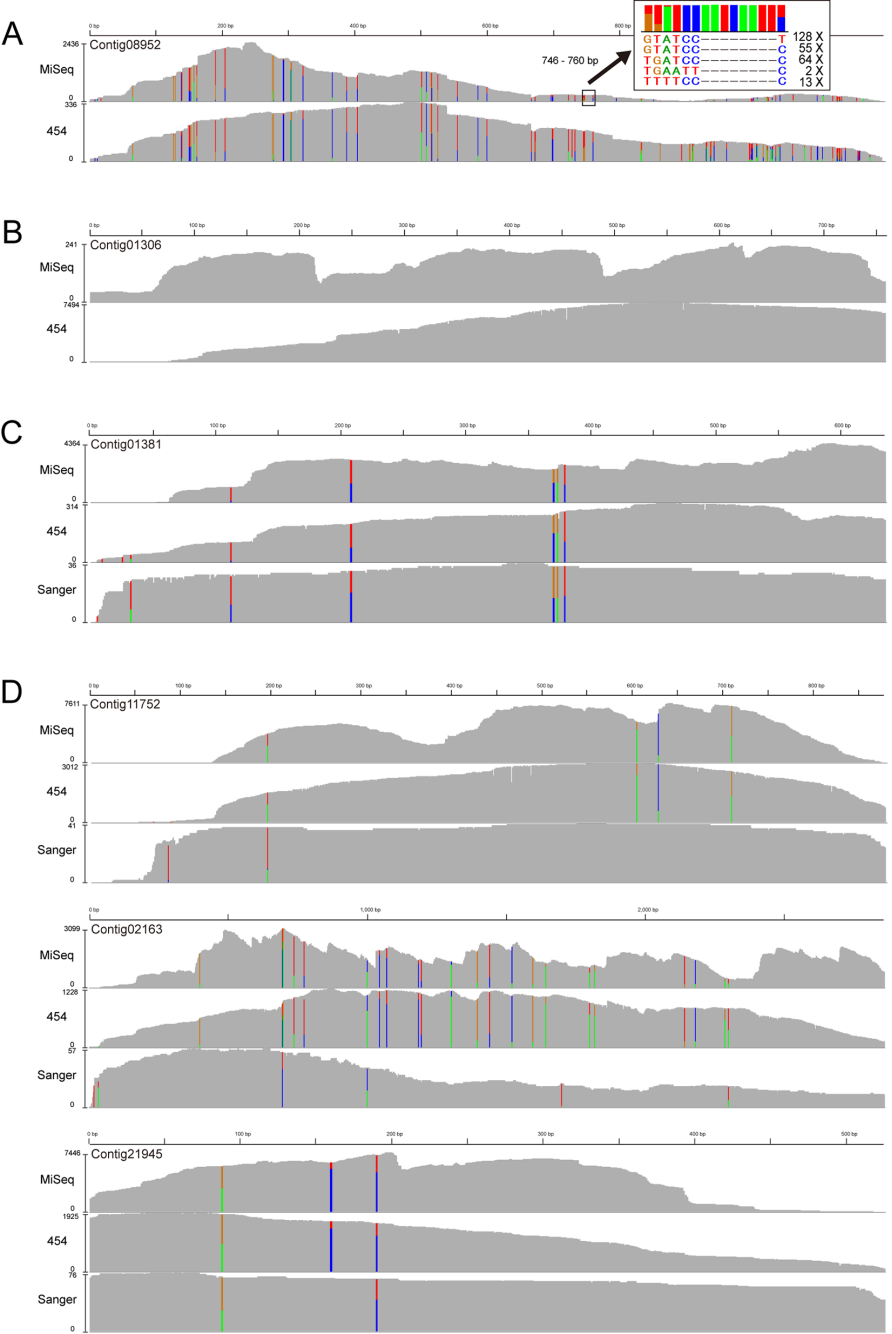
Pattern of SNP frequency and commonality among sequencing platforms. Alignments of MiSeq, 454, Sanger and genome reads against the Reference Gene Model. The colored vertical bars indicate the site of a SNP, and the Y-axis indicates read depth. (A) shows genes with many SNPs, and the top-right image is an enlarged view of a SNP-rich region. SNPs common between MiSeq and 454 are detected for both types of genes. (B) shows genes with no SNPs. Regarding EST sequencing reads produced by the Sanger method, some SNPs are common between MiSeq and 454 (C) but are not detected by Sanger sequencing (D).

Fig 5 shows the MiSeq, 454 and Sanger sequences that were aligned with the Reference Gene Model. The SNP frequency was not constant for all the genes; rather, some genes with many SNPs (A) and some with no SNPs (B) were found. Furthermore, SNPs common to MiSeq and 454 were detected. The top-right image of (A) shows a magnification of an SNP-rich region, showing that there are five different alleles in this region and their ratios vary substantially. While this commonality was similar to that in the EST sequencing carried out by the Sanger method (C), some SNPs were common to MiSeq and 454 but were not detected by Sanger sequencing (D). Although the same strain derived from the same individual was used for the Sanger EST and for MiSeq and 454 sequencing, EST samples had been collected about 10 years earlier than MiSeq and 454 samples.

### ORF Prediction and Codon Usage Analysis

To verify whether the large number of detected SNPs actually results in changes in amino acid sequences of proteins, we first conducted ORF prediction and amino acid translation for each gene. To remove possible frame-shift and mis-assembly sequences, the frames with the longest ORF from the representative sequences of each isogroup were chosen, and the sequences with a post-translation length of 100 aa or longer were considered valid ORFs. As a result, valid ORFs were predicted for 18,677 genes, which accounted for 66.9% of the total isogroups, and their mean amino acid length was 384 aa (1,151 bp). Using this predicted ORF sequence set as a reference, we conducted mutation detection and measured the percentages of amino acid substitutions. Mapping of the MiSeq transcriptome reads to the ORF reference followed by SNP calling showed that 100,302 SNPs, which represented 49.5% of all SNPs detected, caused amino acid substitutions (Table 4). As many as 13,862 genes possessed these non-synonymous SNPs, accounting for 74.2% of the valid ORFs. Furthermore, there were 2,769 short insertion and deletion mutations (InDels) located in a total of 1,998 genes (Table 4).

**Table 4:** Analysis of synonymous and non-synonymous SNPs of ORF sequences.

Mutation	Effect Type	# of mutations	
SNP	Synonymous coding	99064	48.9%
	Non-synonymous coding	100302	49.5%
	Stop gained	3067	1.5%
	Total	202433	
InDel	Codon change plus codon deletion	117	4.2%
	Codon change plus codon insertion	116	4.2%
	Codon deletion	583	21.1%
	Codon insertion	457	16.5%
	Frame shift	1487	53.7%
	Stop gained	9	0.3%
	Total	2769	

To examine the possibility that *D*. *japonica* has codon usage that is especially prone to causing amino acid substitutions due to SNPs, one of three bases constituting each codon was randomly replaced, and pseudo-SNPs were simulated to compute the probability that a non-synonymous amino acid substitution occurs in a completely neutral state. The simulation using the standard codon table for eukaryotes as a control indicated that the probability of the occurrence of an amino acid-substituting SNP was 76.0% (S4 Table). We then conducted the simulation with all the codons constituting the ORFs of *D*. *japonica*, and the results showed that the probability of an amino acid substitution occurring in a completely random state was 79.7% (S4 Table). This value did not differ appreciably from the simulation value obtained with the standard codon table. Actual SNPs within the planarian were not distributed uniformly across all the codon positions, but rather were biased in favor of the third position (53.8%) (S5 Table). This bias reduced the actual amino acid substitution rate compared with the simulation values (Table 4).

### Functional Annotation of Genes that Had Many Mutations

**Fig 6:**
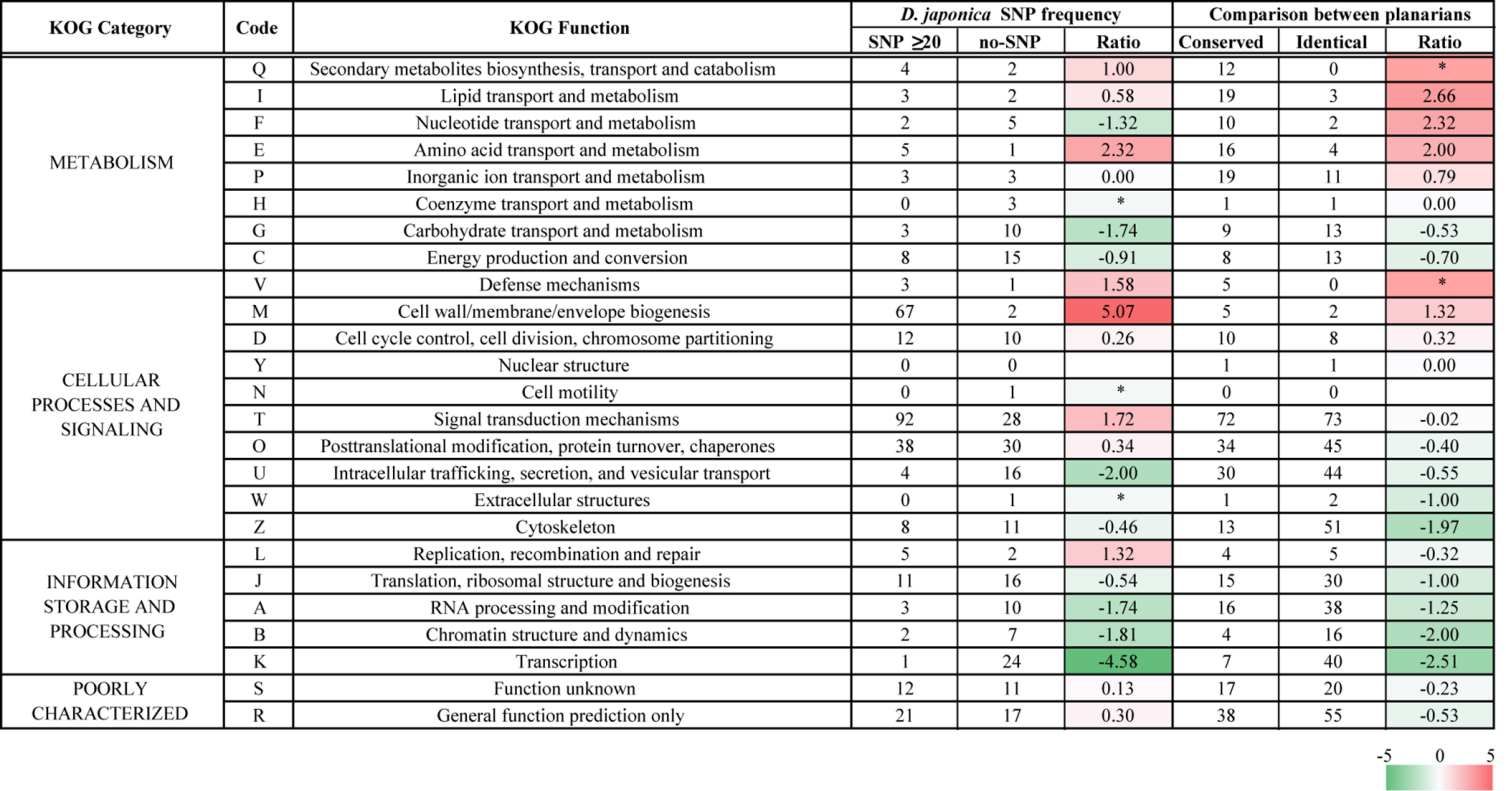
KOG-annotation-based classification of genes that have extremely large numbers of mutations. The genes without any SNPs and the genes with 20 or more SNPs per 1,000 bp were classified regarding KOG function. The heat plot shows the log_2_ SNP ≥20 / no-SNP ratio, with red indicating a high proportion of genes that had an extremely large number of mutations and green indicating that the majority of genes had no SNPs. * indicates a fraction that contained only one of these two types of genes. The right column shows the data of conserved proteins and identical proteins from comparison with a different planarian species (*S*. *mediterranea*). The definitions are derived from the identical match ratio calculation using the amino-acid region conserved between homologous proteins.

Fig 4B shows that many genes had a relatively small number (generally ≤ 10) of SNPs/1000 bp, with a peak value of 4 SNPs/1000 bp. In addition, some genes with no SNPs and some other genes with a very large number of SNPs (≥ 20 /1000 bp) were found, indicating that the number of SNPs differs depending on the gene. Next, we investigated whether there is a specific tendency of the number of SNPs associated with a particular functional category of genes. After each gene was annotated using the NCBI KOG database, the genes were divided into functional categories of KOG [40]. The total number of SNPs was then computed for each gene, and the genes without any SNPs, and those with 20 or more SNPs, per 1,000 bp were extracted from each KOG category. The ratio of these genes was used to test whether there was a difference in the tendency of the number of mutations depending on the gene function. The results revealed that there were many differences in this tendency among functional categories (Fig 6). In this figure, the data shown on the right side are the results from comparison with a different planarian species, *S*. *mediterranea*, reported previously in our EST analysis [18], which were calculated in the same way as the SNP analysis performed here based on the ratio of amino acid substitutions that occur in homologous genes. The two results were correlated in many functions, including “Secondary metabolites biosynthesis, transport and catabolism”, “Amino acid transport and metabolism”, “Defense mechanisms”, “RNA processing and modification” and “Transcription”, but were different in some other categories, namely, “Nucleotide transport and metabolism” and “Signal transduction mechanisms”.

## Discussion

### The Unusual Genome of Planarian

We established asexually reproducing clonal planarians from a single individual, and obtained here a genome sequence of sufficient quality and quantity for *de novo* assembly analysis from this clonal strain. However, although the sequence was subjected to multiple quality control processes and a variety of assembly conditions were also utilized, the lengths of the contigs and the scaffolds obtained thereby were very short. This unusual feature of the planarian genome was also shown by the results of k-mer graph analysis. Neither the boundary between noise/contamination and signal usually observed in whole genome shotgun sequencing nor the heterozygous level represented by a monomodal/bimodal peak was detected with any k-mer value. The presence of a large number of repeat sequences, indicated by high-frequency k-mers, was also confirmed. Taken together, these results suggest that the difficulty of the planarian genome assembly was due to the presence of a very large number of mutations, as well as to the presence of a large number of repeats, which is known to be the most common problem in *de novo* genome assembly.

Because each read sequence generated by next generation sequencers is a clone sequence derived from one genomic DNA fragment, each read directly represents an allele of the chromosome. The example shown in Fig 5A indicates that five alleles (which exceeds the normal heterozygosity) existed, and their ratios and proportions were different. Previously reported studies using comparative analysis of mitochondrial genome sequences showed a great variability in sequence not only between different platyhelminths but also within the same *D*. *japonica* species [41]. Furthermore, there is also a report showing some heterogeneity even within a single individual, as revealed by mutation analysis of COI regions of the mitochondrial genome [42]. However, a single cell contains a large number of copies of the mitochondrial genome, and such coexistence of mutated mitochondrial DNAs in a single cell is commonly known as heteroplasmy. In contrast, our analysis was conducted with the nuclear genome. The results of our analysis do not indicate high heterozygosity between homologous chromosomes in the ancestral planarian which was used to establish the clonal strain, but rather revealed that unique mutations were generated at the cellular level as a result of more than 20 years of asexual reproduction in spite of the population of planarians being completely clonally derived from a single individual. In fact, a large number of mutations were confirmed in the genome sequence of the SSP-9T-5 strain, as in the GI strain (S3 Fig). The SSP-9T-5 strain underwent sexualization and sexual reproduction only once from the GI strain, and subsequently has undergone only asexual reproduction for more than 15 years.

The possibility that the large number of mutations detected in this study represent DNA sequencing errors was ruled out by the following results. The large number of mutations was detected commonly using different sequencing platforms, including GA IIx, MiSeq, 454, and Sanger, and also using different samplings of the genome and RNA (Fig 5). In addition, 454 sequencing is characteristically incapable of accurately determining the number of long homopolymers (such as AAAA and GGGG), but the 454 sequence was used only as a reference to map the MiSeq reads. Differences between MiSeq and 454 are not homozygous SNPs, and were excluded from the analysis. Furthermore, the SNP detection algorithm excludes overlaps of simple sequencing mismatches. The number of SNPs was different depending on the gene, and SNPs were biased to be present in the third position of each codon, presumably as a result of selection pressure (S5 Table).

### Novel Approach for Organisms with a Difficult-To-Decode Genome

A combination of Illumina short reads and an assembly program based on the de Bruijn Graph algorithm is one of the most widely used approaches for *de novo* transcriptome analysis, but in the case of *D*. *japonica*, it failed to adequately deal with the sequence containing an unusually large number of mutations, as was also the case for the *D*. *japonica* genome assembly. In a different planarian species, transcriptome analysis of a clonal strain was carried out with Illumina HiSeq, and it was reported that the contig number did not converge in the assembly using the de Bruijn Graph [43]. A similar problem may have occurred in the present study.

In addition to determining the genome characteristics described above, we attempted to create a reference sequence closer to the full length for *D*. *japonica* by first performing large-scale transcriptome analysis using the Roche 454 system. While the output read number produced was smaller when using 454, this system uses milder mRNA fragmentation conditions (70°C, 30 seconds), can accept a wider range of fragment size (900–1,000 bp, Table 1), and can sequence longer reads than Illumina MiSeq. Therefore, the output regions are less biased in the gene, the error rate is also low, and the system is superior in reconstructing the full-length gene sequence [44]. To compensate for the low quantity of the 454 output, we conducted large-scale sequencing, and used the overlap-consensus algorithm in the assembly process, which is useful for assembling long reads, although it has a higher computing cost. As a result, we were able to absorb the large number of SNPs and InDels between the sequences and to successfully obtain a high-quality consensus sequence.

To enable accurate analyses of gene expression levels and mutations even in organisms in which the genome sequence has not been determined, we thus propose the model we report here as a new model that we call the Reference Gene Model, which uses a transcriptome assembly and a contig graph for its production, and is a virtual genome sequence. The results of our differential gene expression analysis demonstrated that the Reference Gene Model has high accuracy and enables quantitative transcriptome analysis even for an organism that has an extremely large number of mutations. Furthermore, many anterior blastema specific genes had hits of uncharacterized proteins with high levels of homology, suggesting the possibility that there are many new head regeneration factors yet to be characterized in detail. Thus, differential gene expression and mutation analyses have now become possible using *D*. *japonica*. The Reference Gene Model will be useful for many future studies.

### Mutation Analysis

Surprisingly, our large-scale transcriptome analysis confirmed that extremely high numbers of mutations were located not only outside the gene regions but also in the coding regions of the genes. Approximately half of the SNPs were found to be non-synonymous mutations. Our ORF analysis and mutation simulation revealed that *D*. *japonica* did not have a special codon usage resistant to mutation. However, the actual substitution rate was lower than the simulated values because of the SNP bias to the third position of the codon, suggesting that some sort of selection pressure was applied to mutations.

In our previous report, we demonstrated that the degree of amino acid substitutions between two planarian species was different for each functional category, and the substitutions were particularly abundant in categories related to environmental adaptation [18]. Also in the present analysis, the degree of number of mutations was clearly different for different functional categories of genes, and the majority of the present differences were in accord with the results from our previous comparison between different planarian species. This accordance supports our previously reported hypothesis that the potential for changes is different in genes required to respond to changes in the external environment versus genes that are not so required. The genes of the “Defense mechanisms” and the “Signal transduction mechanisms” function classes contained a large number of mutations, particularly in genes involved in antiviral responses and in regulation of apoptosis. In addition, genes with many mutations were also detected in the function class “Replication, recombination and repair”, and it could be speculated that the high mutation rates of these genes might contribute to the unusually large number of mutations in the planarian. Interestingly, despite the presence of such a large number of mutations that are expected to alter protein structures and to have a significant impact on life activities, no abnormalities have been observed in our cultured planarians [45–47].

In higher organisms, accumulation of mutations is a major factor that may cause a cell to become a cancer cell, but cancer is rarely found in invertebrates in nature, and there have been no reports of cancer in planarians in the natural state. Tumor induction by artificial means such as administration of a carcinogen or ionizing radiation has been attempted in numerous studies, but outcomes that can be clearly identified as cancer have not been obtained [48, 49]. Interestingly, homologs of mammalian tumor suppressor genes such as *p53* and *PTEN*, however, have been found in planarians, and inhibition of their functions with RNAi has been reported to cause abnormal proliferation of stem cells, leading to lethality [50, 51]. Furthermore, recent research indicates that innately asexual planarians maintain their telomere length during cell division, whereas sexual worms show shortening of their telomeres [52]. Planarians might possess a mechanism of stem cell control that prevents the production of cancerous cells and the death of planarians even in a state in which a large number of mutations have been accumulated.

### Acquisition of Genetic Diversity during Asexual Reproduction

In theory, loss of sexual reproduction is considered an evolutionary disadvantage because genetic diversity is not obtained through recombination. However, the bdelloid rotifer lineage *Philodina roseola* has evolved for tens of millions of years without sexual reproduction [53]. Gene copies of *P*. *roseola* showed different structures and functions resulting from divergence of former alleles, and this suggests a hypothesis that the newly obtained genes play complementary roles in survival [53–55]. The results indicate that genetic diversity could be acquired even in asexual reproduction if an organism evolved a mechanism to accumulate mutations in individual genomes. Because stem cells generally undergo cell division at high frequency and are morphologically and functionally undifferentiated, they are susceptible to environmental stresses, such as radiation, and are prone to mutations. Planarian’s neoblasts account for as many as 30% of the total cells. This very large population might in part account in the following way for the extremely large number of mutations we found here: the neoblasts contribute to the planarian's outstanding regenerative capacity, and are also known to be involved planarian's self-propagation (asexual reproduction) and body homeostasis, and to give rise to germline cells during sexualization [56–58]. While genetic diversity is acquired in sexual reproduction through recombination between paternal and maternal genes during gametogenesis, this is not possible in asexual reproduction. Even in asexual reproduction, new traits can be acquired on a cell-by-cell basis through mutations, but most of these mutations are probably eliminated at the single cell level in the course of homeostasis or asexual reproduction. However, some non-fatal mutations could propagate in a planarian's body via proliferation of mutation–possessing stem cells. Thus, genetic diversity could be acquired at the level of a single individual planarian. However, interestingly, the planarian species studied here, *D*. *japonica*, can switch its reproduction system from asexual to sexual. If the mutation–possessing stem cells differentiate into germ cells and develop to produce adult planarians after fertilization, these mutated genes would be transmitted to the next generation and some of them could become fixed and easily propagated in their colonies by asexual reproduction, if these mutated phenotypes were adaptive in the new circumstances. Therefore, it is conceivable that via asexual reproduction, planarians can pass on to the next generation mutations accumulated in their neoblasts, which may then be contributed to the germline upon sexualization. The results of this study thus suggest the possibility that planarians can acquire genetic diversity by asexual-sexual cycling reproduction.

## Conclusion

The k-mer analysis of the genome sequence showed neither homozygous nor heterozygous characteristics, and the *de novo* assembly also produced very small contigs/scaffolds. Similar results were also obtained from the transcriptome analysis. To accomplish quantitative mutation analysis of this organism, a new gene reference model was constructed. A very large number of SNPs, insertions, and deletions were detected in the coding regions by comprehensive mutation analysis, about half of which would cause amino acid substitutions. Surprisingly, despite such a large number of mutations, no abnormalities have been observed in our clonal planarians. The level of mutations was not constant across all genes, and this result was in accord with our previously reported findings regarding amino-acid substitutions that had occurred between different planarian species. During asexual reproduction, planarians might accumulate genetic diversity in their bodies as a result of mutations, and some mutant stem cells adaptive for a planarian's environmental circumstances might proliferate inside their bodies. If these stem cells are converted to germline stem cells in the course of sexualization and then participate in fertilization, they could become fixed in the genotype of the next generation. The results of the present study thus provide a new insight into the possible evolutionary significance of asexual reproduction in planarians.

## Materials and Methods

### Planarian Resources

The planarian *Dugesia japonica* was collected from the Iruma River in Gifu prefecture, Japan, in 1990. A clonal strain, GI, which was derived from one single such planarian, was maintained asexually in autoclaved tap water at 22–24°C in dim light. The worms were fed raw chicken liver twice a week, and increased in number by fission and regeneration approximately every 2 weeks. Another clonal strain, SSP, which was derived from a single animal of the GI strain, underwent sexualization in 1994, and has since then been maintained asexually in the same way as the GI strain. In June 2005, the SSP strain was re-cloned from a single animal; we call this strain SSP-9T-5 (S4 Fig). The planarian *Schmidtea mediterranea* from the CIW4 clonal strain was cultured at 20°C in artificial freshwater solution B5282 (Sigma) [59], and was fed raw beef liver twice weekly. In all experiments, planarians of 8- to 10- mm length that had been starved for at least 1 week were used. No specific permissions were required for the locations/activities in this study, and this study did not involve endangered or protected species.

### Fluorescence-Activated Cell Sorting (FACS) Analysis

Preparation of dissociated planarian cells and flow cytometry analyses were performed using slight modifications of protocols previously described [60, 61], which have high resolution that can distinguish between G1 and G2/M phase in live cells. Five worms per sample were collected in December 2003, and were dissociated into single cells with 0.1% (w/v) trypsin in 5/8 Holtfreter's solution at 20°C for 5 min. The dissociated cells were stained at 20°C for 2 hours with 18 μg/mL Hoechst 33342 (Sigma) for estimation of DNA content and with 0.5 μg/mL Calcein AM (Dojindo) for estimation of cell size. To eliminate dead cells during the measurement, 1 μg/mL propidium iodide (Dojindo) was added to the dissociated cells. All flow cytometry data were acquired using a BD FACSVantage SE (Becton Dickinson) and analyzed using FlowJo software Macintosh version 8.1.1 (Tree Star).

### Genome and RNA Resources

Genomic DNA was extracted from 200 adult planarians derived from clonal strain SSP-9T-5 in July 2009. The planarians were immediately frozen with liquid nitrogen, and then crushed with a mortar and pestle. The resultant planarian cell powder was lysed with Nuclei Lysis Solution (Promega) including proteinase K at 60°C for 1 hour. After RNaseA treatment, proteins were removed using Protein Precipitation Solution treatment (Promega), and the genomic DNA was precipitated with isopropanol and resuspended in TE buffer.

The GI strain was used for transcriptome analysis. To extract total RNA, head pieces (*n* ≥ 800) were collected from planarians transversely amputated anterior to the pharynx in July 2008 (S5 Fig). Pieces of the anterior or posterior ends, which included regenerating blastemas, were collected from the fragments (*n* ≥ 300) of planarians that had been transversely amputated posterior to the pharynx (S5 Fig) in April 2010. Total RNA was extracted from each of these pools of tissues using ISOGEN-LS (Nippon Gene), and then subjected to two rounds of polyA-plus RNA enrichment (purification) with an Oligotex-dT30 Super mRNA Purification Kit (Takara Bio) following the manufacturer’s protocols. RNA quantities were determined using a Nanodrop spectrophotometer (Thermo Scientific) and a Quant-iT RiboGreen RNA Assay Kit (Invitrogen), and their qualities were assessed using an Agilent 2100 Bioanalyzer (Agilent Technologies). The genomic DNA and RNA resources did not undergo pre-amplification before library preparation.

### Genome Sequencing

To construct different insert-size libraries, genomic DNA was separated by agarose gel electrophoresis, and regions containing DNA of about 300 bp, 350 bp and 400 bp length were cut out by reference to their corresponding size markers. Libraries for genomic DNA sequencing were prepared using a Paired-End DNA Sample Prep Kit (Illumina) according to the manufacturer's instructions. The resultant libraries were sequenced (2 x 150 cycles paired-end) on an Illumina GAIIx instrument.

### Genome K-Mer Frequency Analysis and Assembly

The genome sequence of *S*. *venezuelensis* was obtained from the DDBJ database [DDBJ:DRA000971] as a control. To estimate the best k-mer value for the genome analysis, the KmerGenie ver. 1.6476 program was executed with k-mer range from 21 to 121. We prepared three types of genome sequence dataset. For a valid paired-end dataset, Trim Galore ver. 0.3.1 was used to remove adapters and low-quality sequences of genome sequences using -e 0.1 -q 20 parameters [62]. To make a merged long read from paired-end Illumina reads that overlapped at their 3’ ends, we used the SeqPrep program with -q 20 option. Quality cut and adapter trimming were performed at the same time. All of the merged-read and the unmerged-read pairs were included in the k-mer and assembly analysis. The error correction dataset was constructed using SOAPec ver. 2.01 [63] based on the k-mer frequency spectrum (KFS) algorithm. The k-mer value was 27, and the valid paired-end sequences were used as initial dataset. In every preparation, orphan sequences, with only one direction remaining as a result of the filtering process, were excluded from further analyses. The k-mer frequency analysis of the whole genome was performed using the Trim Galore-treated genome sequences.

Next, two assembly programs, SOAPdenovo ver. r240 and Platanus ver. 1.2.1, were used for *de novo* assembly of three types of sequences. In the case of SOAPdenovo, k-mer values of 61 and 83, which were determined by KmerGenie analysis, were used for the valid paired-end reads and merged long reads, respectively. Platanus was run with -u 0.3 parameter for a high heterozygosity genome. The statistics of assembly results, the number of contigs/scaffolds, the N50 values, and the average lengths were calculated using length over 100 bp.

### Transcriptome Sequencing

cDNA libraries for sequencing were constructed using a GS FLX Titanium Rapid Library Preparation Kit (Roche Applied Science) according to the manufacturer’s instructions. DNA sequencing was performed using a Roche 454 GS FLX and FLX+ platform with Titanium chemistry (Roche Applied Science) using GS FLX PicoTiterPlates with the large region gasket according to the manufacturer’s instructions. For transcriptome sequencing using Illumina MiSeq, cDNA libraries were constructed using a TruSeq RNA Sample Prep Kit v2 (Illumina). The cDNA libraries were sequenced (251 cycles of paired-end) on MiSeq using a MiSeq Reagent Kit v2 (Illumina) according to the manufacturer’s protocols.

### Transcriptome Assembly and Reference Gene Model

Raw 454 transcriptome reads were trimmed using a function of Newbler assembler ver. 2.6 (Roche). The valid 454 reads were assembled using the Newbler assembler with default parameters. The Standard Flowgram Format (SFF) files of the sequence, which contained the original flow-based signal trace, were used as input to the assembly process of Newbler. The assembly process took approximately 3 months using a workstation that had four Intel Xeon 1.87 GHz CPUs (total 48 cores) and 512 GB main memory. Quality and adapter trimming of raw MiSeq transcriptome sequences were done using Trim Galore with parameters -e 0.1 -q 20. To perform *de novo* transcriptome assembly, MiSeq and MiSeq-454 hybrid valid data were assembled by Trinity ver. r20131110 using default parameters.

The Reference Gene Model was constructed using only 454 assembly results. The assembled contigs in each isogroup were ordered and connected based on the contig-graph information created by Newbler to make virtual genome sequences without introns. A representative sequence from an isogroup was chosen as an isotig sequence with the best BLAST score against NCBI NR database release 2013-11-13 using the BLASTX program. The representative sequences were annotated based on the best hit in the UniProt-SwissProt and -TrEMBL database release 2013–10 using BLASTX with threshold 1e-5. Additionally, the annotation of the representative sequence was employed as an annotation of each respective isogroup.

### Differential Gene Expression Analysis

AB and PB valid forward reads of MiSeq were trimmed to the first 200 bp starting from the 5’ end. Then, only 5’ trimmed reads were mapped onto the Reference Gene Model using bowtie2 program ver. 2.1.0 with the local-alignment option [64]. After counting mapped reads categorized according to isogroup, genes differentially expressed between AB and PB were identified by DEGseq ver. 1.16.0 of the BioConductor package using the loess normalization method and likelihood ratio test. The threshold was set at minimum read count of 10 or more for AB and PB, *p*-value < 0.001 and having an AB/PB fold change of 2 or more, and being hit with a BLAST E-value of 1e-5 or less against the TrEMBL database.

### Reverse Transcription and Quantitative RT-PCR Analysis

Total RNAs from 50 AB and PB fragments each were isolated using ISOGEN-LS (Nippon Gene) in May 2011. First-strand cDNA was synthesized using a QuantiTect Reverse Transcription Kit (QIAGEN). The mixture for quantitative RT-PCR analysis contained 1x QuantiTect SYBR green PCR master mix (QIAGEN), 0.3 μM of each gene-specific forward and reverse primer, and an appropriate dilution of the synthesized cDNA. Quantitative analysis of the amount of each gene product was carried out as previously described [65] using a Thermal Cycler Dice Real Time System II (Takara Bio). The conditions of PCR were as follows: first, incubation at 50°C for 2 minutes, second, incubation at 95°C for 15 minutes, and then 45 repeats of the following 3 steps: incubation at 95°C for 15 seconds, at 55°C for 30 seconds and at 72°C for 1 minute. Measurements were normalized by the expression level of a constitutively transcribed housekeeping gene, GAPDH. The mean of three replicate qRT-PCR assays was reported. Oligonucleotide primer sequences used for the assays are listed in S6 Table.

### SNP Detection

The valid MiSeq paired-end reads of the AB, PB and HP libraries were trimmed to the first 200 bp from the 5’ end to increase the accuracy of the detection of mutations, and then were mapped onto the Reference Gene Model using the BWA program ver. 0.7.4-r385 using the BWA-MEM algorithm [66]. The valid reads of the genome were mapped using the same method as used in MiSeq. SNPs and InDels were called using the Unified Genotyper in the Genome Analysis Toolkit (GATK) framework ver. 2.8–1 after applying local realignment. The filter function of SnpSift ver. 3.4e [67] including the SnpEff package [68] was used to determine whether a mutation genotype was homozygous or heterozygous, and allelic fraction and local read depth of alleles were estimated using the mpileup command of samtools ver. 0.1.19 [69]. To remove the heterozygosities that the ancestral individual originally possessed, only SNPs with a SNP rate of 0.06–0.30 or 0.70–0.94 were analyzed. Furthermore, to enhance the data reliability, only SNPs with a minimum read depth ≥3 were included in the analysis. A total of 54,752 *D*. *japonica* EST sequences were obtained from the DDBJ database (FY925127—FY979285 and AK388576—AK389168). For comparison with the MiSeq results, 454 and Sanger EST sequences were mapped and realigned using the same method as used for MiSeq analysis. The alignments were visualized using the Integrative Genomics Viewer (IGV) ver. 2.3.25 [70] for determination of correlations among sequencing platforms, and among dates of sampling.

### ORF Prediction and Codon Usage

Before the detection of non-synonymous mutations in each gene, the longest ORF of the representative sequence was predicted by using the Galaxy get_orfs_or_cdss script with minimum amino-acid length 100 to obtain high accuracy and long reference sequences [71]. A codon usage table of the reference sequence was created using the cusp of EMBOSS package ver. 6.4.0.0 [72]. Mapping and mutation calling using the ORF sequences were performed using the same method as used for MiSeq mutation analysis, and the SnpEff program was used to classify SNPs and InDels based on the structural effects of the amino acid sequence.

### Gene Classification Based on KOG Annotation

All gene sequences were BLASTed against NCBI KOG database release 2014-02-12 using threshold 1e-10, and all genes with hits were classified into KOG category and function. Next, genes with 20 or more SNPs per 1000 bp and genes with no SNPs were filtered from representative sequences over 600 bp long. The SNP filter conditions used were chosen as the same as used for SNP detection, and transposon genes were discarded.

## Supporting Information

S1 Fig All k-mer spectra of the *D*. *japonica* genome. (TIF)

S2 Fig Differential gene expression analysis and qRT-PCR validation performed using the Reference Gene Model. Differential gene expression analysis and qRT-PCR validation performed using the Reference Gene Model. (A) Mapping results of MiSeq trimmed reads against the Reference Gene Model. (B) A boxplot of read counts for each gene. (C) MA plot of AB vs PB. Y-axis represents the intensity ratio, and X-axis represents the intensity for each transcript. The red points identify genes differentially expressed between AB and PB by the MA-plot-based method with a random sampling model. (D) qRT-PCR validation of RNA-seq data for 17 genes.
(TIF)

S3 Fig Genome sequence alignment of Dj-SSP strain. (TIF)

S4 Fig Summary of the *D*. *japonica* lineage and sample preparation. (TIF)

S5 Fig Sampling scheme of the planarian RNA resources. (TIF)

S1 Table Summary of *D*. *japonica* genome sequence. (PDF)

S2 Table Statistics of *de novo* genome assembly. (PDF)

S3 Table List of differentially expressed genes in anterior blastema. (PDF)

S4 Table Random SNP simulation of codon usage between standard and *D*. *japonica*. (PDF)

S5 Table Codon position bias of SNPs. (PDF)

S6 Table Primer sequences for quantitative RT-PCR analysis. (PDF)
