# Supplementary figures and images for: Unusually Large Number of Mutations in Asexually Reproducing Clonal Planarian Dugesia japonica
Source: PLoS One. 2015 Nov 20;10(11):e0143525. doi: 10.1371/journal.pone.0143525 (PMC4654569; doi:10.1371/journal.pone.0143525)

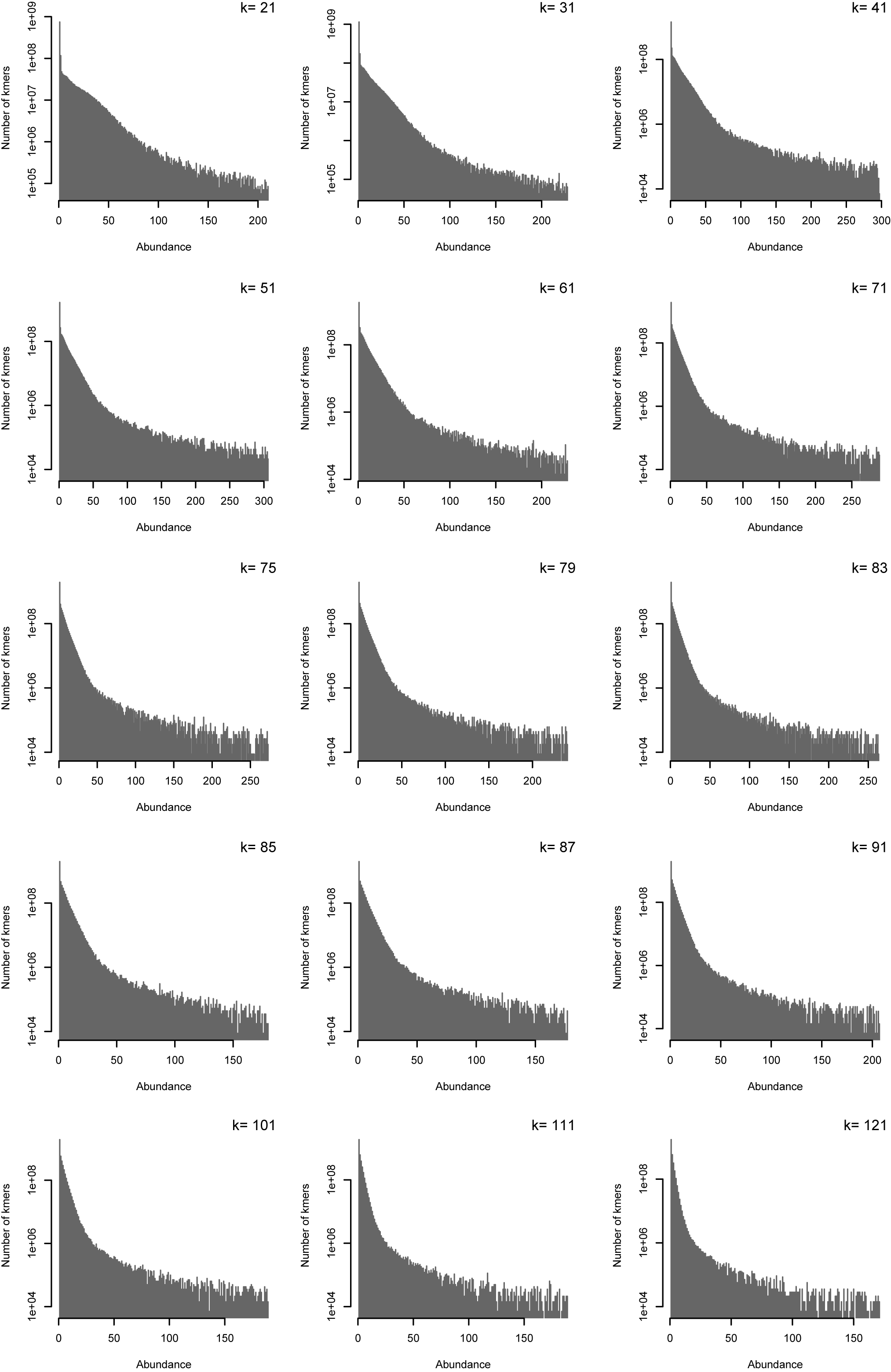

Supplement: S1 Fig — (TIF) [file pone.0143525.s001.tif]

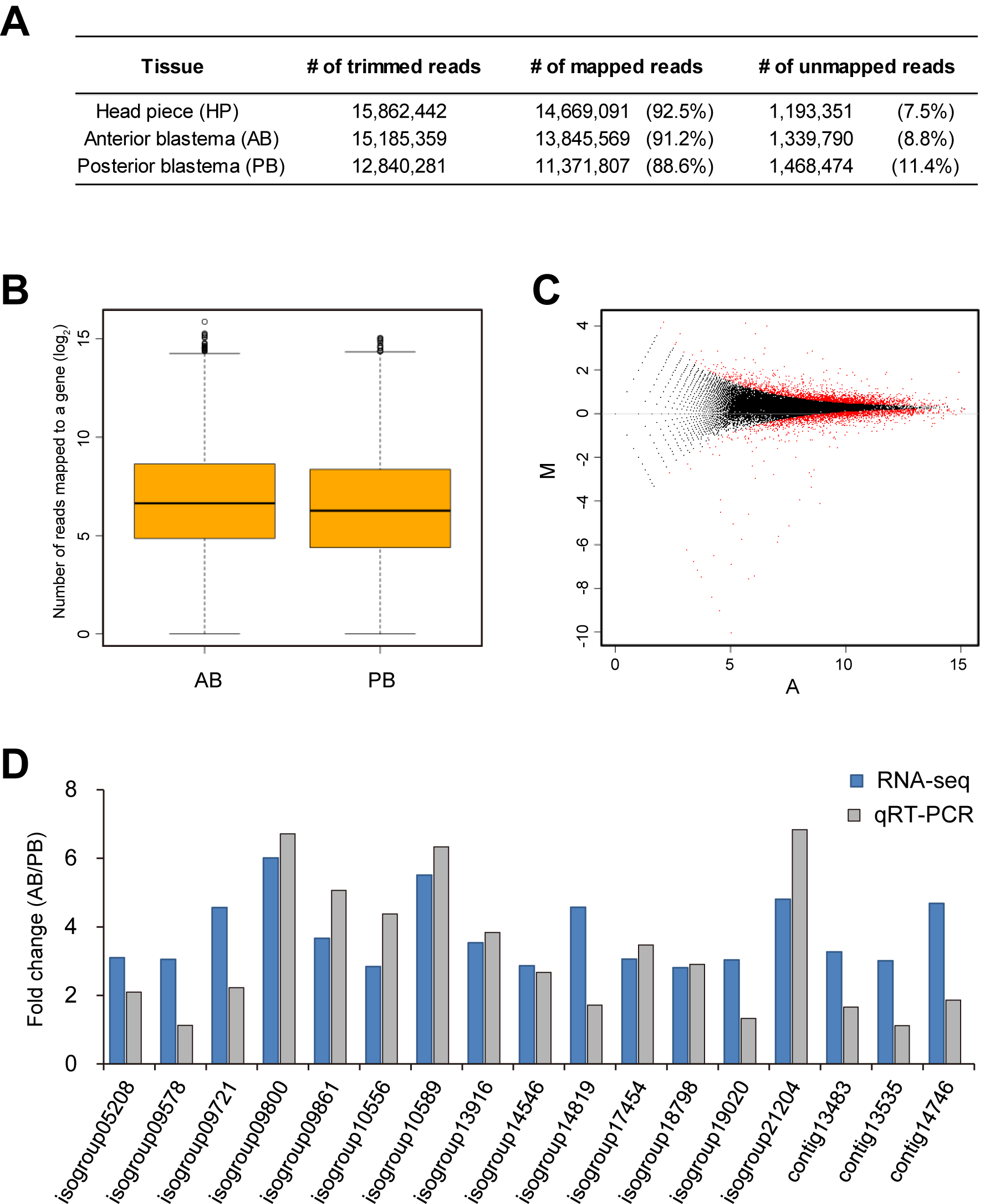

Supplement: S2 Fig — Differential gene expression analysis and qRT-PCR validation performed using the Reference Gene Model. (A) Mapping results of MiSeq trimmed reads against the Reference Gene Model. (B) A boxplot of read counts for each gene. (C) MA plot of AB vs PB. Y-axis represents the intensity ratio, and X-axis represents the intensity for each transcript. The red points identify genes differentially expressed between AB and PB by the MA-plot-based method with a random sampling model. (D) qRT-PCR validation of RNA-seq data for 17 genes. (TIF) [file pone.0143525.s002.tif]

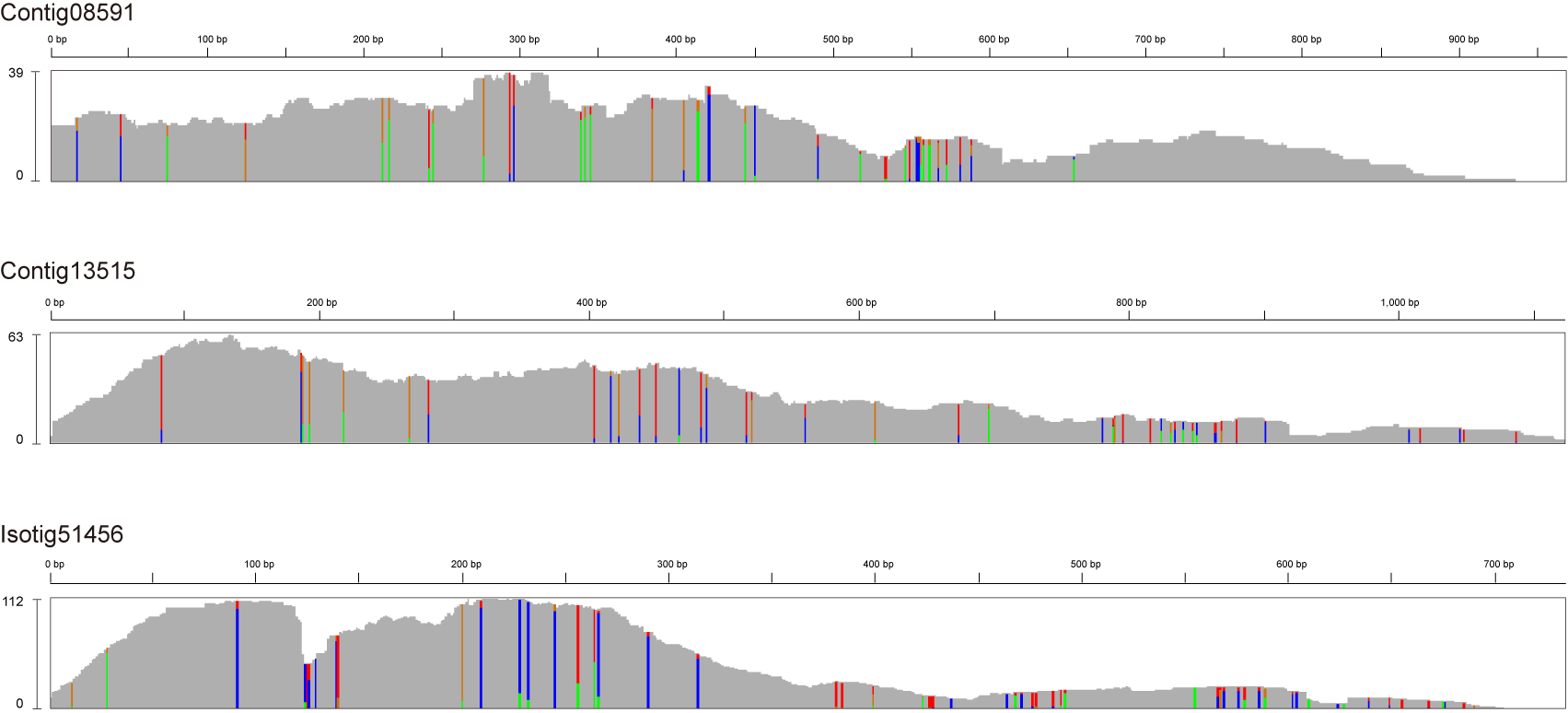

Supplement: S3 Fig — (TIF) [file pone.0143525.s003.tif]

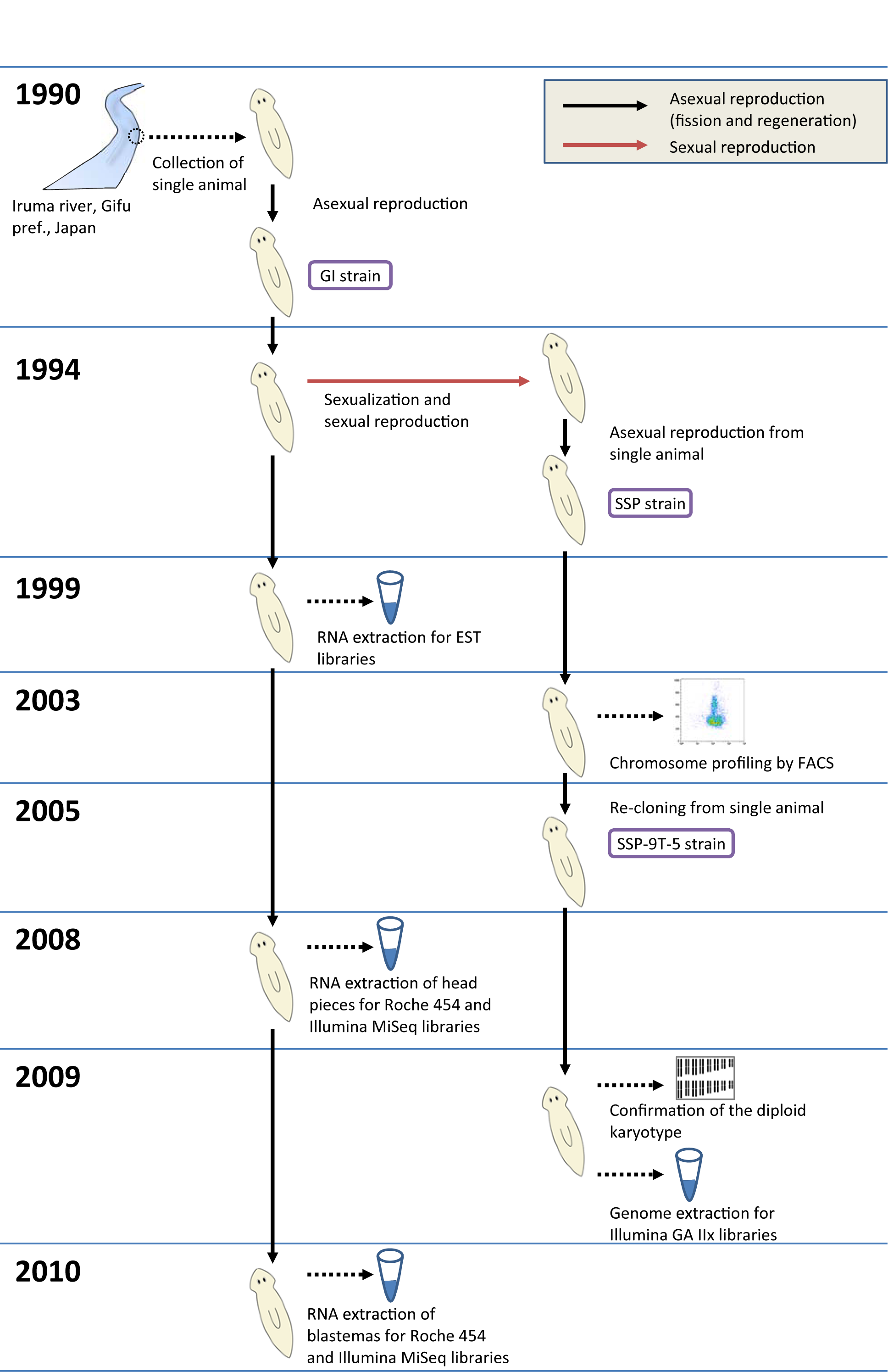

Supplement: S4 Fig — (TIF) [file pone.0143525.s004.tif]

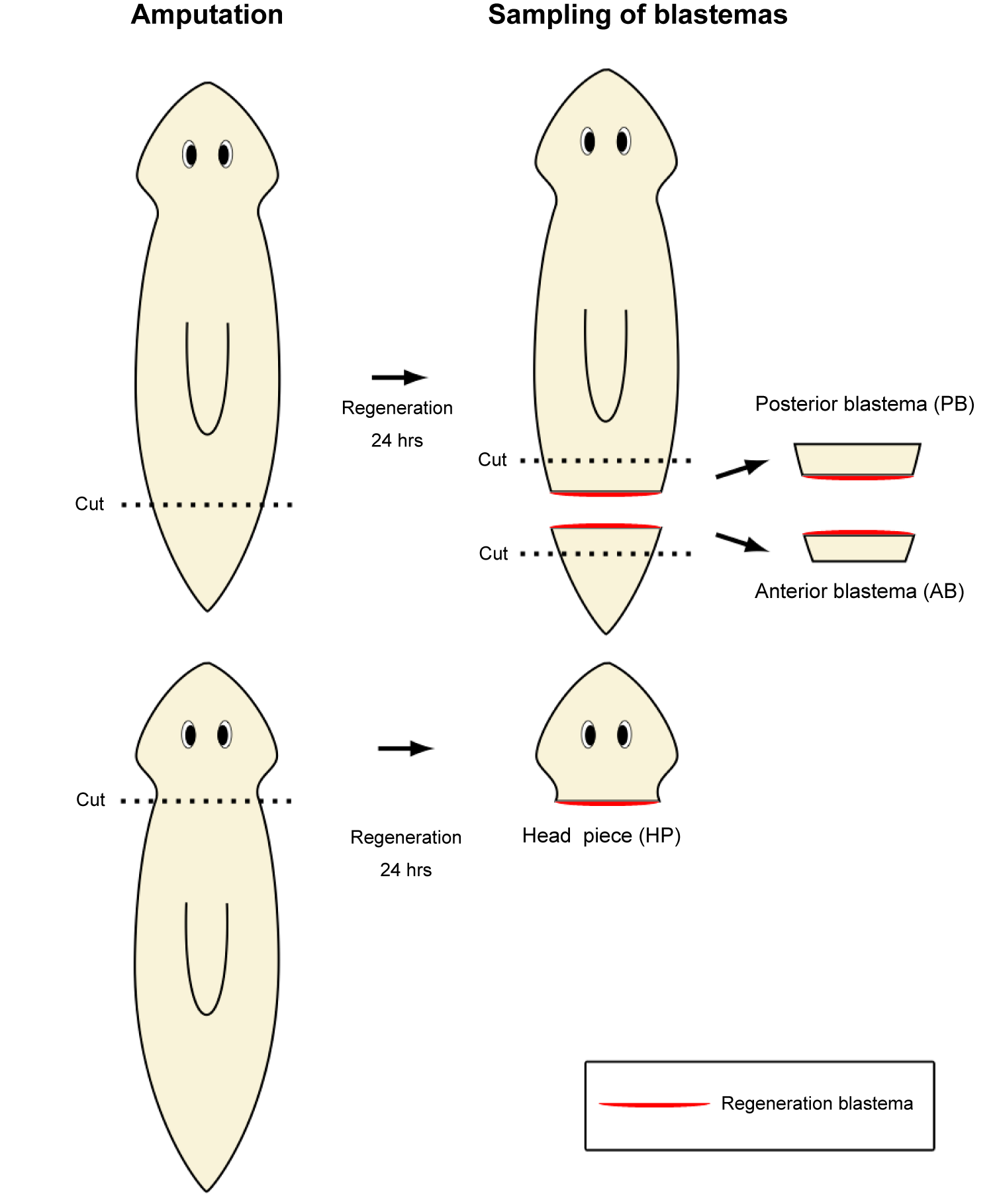

Supplement: S5 Fig — (TIF) [file pone.0143525.s005.tif]
