# Supplementary material for: Unusually Large Number of Mutations in Asexually Reproducing Clonal Planarian Dugesia japonica
Source: PLoS One. 2015 Nov 20;10(11):e0143525. doi: 10.1371/journal.pone.0143525 (PMC4654569; doi:10.1371/journal.pone.0143525)
Supplement: S1 Table — (PDF) [file pone.0143525.s006.pdf]

Quality control	Library insert size	Read type	# of reads	Total number of reads (bp)	Estimated genome coverage
**Raw reads**	300	Forward	126,603,871	18,990,580,650	
		Reverse	126,603,871	18,990,580,650	
	350	Forward	88,461,684	13,269,252,600	
		Reverse	88,461,684	13,269,252,600	
	400	Forward	82,558,738	12,383,810,700	
		Reverse	82,558,738	12,383,810,700	
	Total		595,248,586	89,287,287,900	99 ×
**Quality-value based trimming**	300	Forward	124,661,428	17,291,041,760	
		Reverse	124,661,428	14,478,229,955	
	350	Forward	87,011,210	11,959,962,617	
		Reverse	87,011,210	9,876,951,659	
	400	Forward	81,260,121	11,461,463,022	
		Reverse	81,260,121	9,253,940,172	
	Total		585,865,518	74,321,589,185	83 ×
**Overlapping and merging**	300	Merged	111,634,593	19,570,236,503	
		Forward orphan	14,677,997	2,201,699,550	
		Reverse orphan	14,677,997	2,201,699,550	
	350	Merged	71,103,678	14,545,577,717	
		Forward orphan	17,154,993	2,573,248,950	
		Reverse orphan	17,154,993	2,573,248,950	
	400	Merged	43,723,953	10,702,734,213	
		Forward orphan	38,797,894	5,819,684,100	
		Reverse orphan	38,797,894	5,819,684,100	
	Total		367,723,992	66,007,813,633	73 ×
**Error correction**	300	Corrected forward	120,995,614	16,779,166,011	
		Corrected reverse	120,995,614	14,067,060,179	
		Orphan	3,229,535	354,445,348	
	350	Corrected forward	84,082,649	11,578,063,331	
		Corrected reverse	84,082,649	9,588,429,873	
		Orphan	2,580,352	284,044,918	
	400	Corrected forward	78,872,623	11,112,113,980	
		Corrected reverse	78,872,623	8,996,649,065	
		Orphan	2,188,531	250,279,452	
	Total		575,900,190	73,010,252,157	81 ×
