# Supplementary material for: Unusually Large Number of Mutations in Asexually Reproducing Clonal Planarian Dugesia japonica
Source: PLoS One. 2015 Nov 20;10(11):e0143525. doi: 10.1371/journal.pone.0143525 (PMC4654569; doi:10.1371/journal.pone.0143525)
Supplement: S2 Table — (PDF) [file pone.0143525.s007.pdf]

Quality control	Assembler	Parameter	Contig N50 (bp)	Total contig length (bp)	Scaffold N50 (bp)	Total scaffold length (bp)
Quality-value based trimming	SOAPdenovo	k-mer=61	178	2,707,596,023	220	2,827,016,379
Overlapping and merging	SOAPdenovo	k-mer=83	222	3,677,151,163	226	3,684,100,940
Error correction	SOAPdenovo	k-mer=83	233	289,672,692	273	294,563,714
	Platanus	u=0.3	114	4,419,099,506	290	954,639,547

* For each assembly, only contigs/scaffolds ≥ 100 bp in length are shown
