# Supplementary material for: Unusually Large Number of Mutations in Asexually Reproducing Clonal Planarian Dugesia japonica
Source: PLoS One. 2015 Nov 20;10(11):e0143525. doi: 10.1371/journal.pone.0143525 (PMC4654569; doi:10.1371/journal.pone.0143525)
Supplement: S3 Table — (PDF) [file pone.0143525.s008.pdf]

Isogroup	Length (bp)	Read count of AB	Read count of PB	Normalized fold change (log_2)	*P*-value	SWISS-PROT annotation	E-value	TrEMBL annotation	E-value	NCBI NR annotation	E-value
isogroup06094	1631	212	12	3.86	0	Sodium-dependent phosphate transporter 1 OS=Xenopus tropicalis GN=slc20a1 PE=2	8.00E-68	Uncharacterized protein OS=Lottia gigantea GN=LOTGIDRAFT_239412 PE=4 SV=1	1.00E-159	hypothetical protein LOTGIDRAFT_239412 [Lottia gigantea]	1.00E-159
isogroup13102	1454	240	19	3.38	0	Fibroblast growth factor receptor-like 1 OS=Homo sapiens GN=FGFRL1 PE=1 SV=1	8.00E-09	NDK-6 OS=Dendrocoelum lacteum PE=2 SV=1	1.00E-121	Smed-NDK-3 [Schmidtea mediterranea]	6.00E-28
isogroup19006	874	256	32	2.72	0	Transferrin receptor protein 1 (Fragment) OS=Rattus norvegicus GN=Tfrc PE=2 SV=1	0.63	Cell wall integrity and stress response component 1 OS=Schistosoma japonicum PE=2 SV=1	1.00E-43	Cell wall integrity and stress response component 1 precursor [Schistosoma japonicum] emb|CAX77492.1| Cell wall integrity and stress response component 1 precursor [Schistosoma japonicum]	9.00E-44
isogroup09800	2633	995	136	2.59	0	Fibroblast growth factor receptor-like 1 OS=Homo sapiens GN=FGFRL1 PE=1 SV=1	2.00E-04	Nou darake like-5 OS=Schmidtea mediterranea PE=2 SV=1	0	nou darake like-5 [Schmidtea mediterranea]	0
isogroup05269	1804	844	125	2.47	0	Sodium- and chloride-dependent glycine transporter 2 OS=Rattus norvegicus	1.00E-176	Transporter (Fragment) OS=Lottia gigantea GN=LOTGIDRAFT_104703 PE=3 SV=1	0	hypothetical protein LOTGIDRAFT_104703, partial [Lottia gigantea]	0
isogroup10589	2094	228	34	2.46	0	Fibroblast growth factor receptor-like 1 OS=Gallus gallus GN=FGFRL1 PE=1 SV=1	2.00E-19	Smed-NDK-4 (Fragment) OS=Schmidtea mediterranea PE=2 SV=1	0	Smed-NDK-4 [Schmidtea mediterranea]	0
isogroup21204	742	117	20	2.26	2.30E-14	Protein lozenge OS=Drosophila melanogaster GN=lz PE=2 SV=2	1.00E-28	Runt-1 OS=Schmidtea mediterranea PE=2 SV=1	1.00E-107	runt-like 1 protein [Schmidtea mediterranea] gb|AFJ24721.1| runt-1 [Schmidtea mediterranea]	1.00E-107
contig14746	1270	1231	216	2.23	0	Cholesterol 24-hydroxylase OS=Mus musculus GN=Cyp46a1 PE=2 SV=1	1.00E-42	Predicted protein OS=Nematostella vectensis GN=v1g236002 PE=3 SV=1	5.00E-56	PREDICTED: cholesterol 24-hydroxylase-like [Columba livia]	5.00E-59
isogroup14819	1223	89	16	2.19	6.80E-11	Putative zinc finger protein 840 OS=Homo sapiens GN=ZNF840 PE=5 SV=5	1.00E-06	Zinc finger protein 40 OS=Echinococcus granulosus GN=EgrG_000673000 PE=4 SV=1	9.00E-54	zinc finger protein 40 [Echinococcus granulosus]	6.00E-54
isogroup09721	2698	150	27	2.19	0	Sodium-dependent noradrenaline transporter OS=Mus musculus GN=Slc6a2 PE=2 SV=2	4.00E-37	Transporter OS=Crassostrea gigas GN=CGI_10022090 PE=3 SV=1	3.00E-39	Sodium- and chloride-dependent glycine transporter 2 [Crassostrea gigas]	2.00E-39
isogroup26183	111	153	30	2.07	1.11E-16	Uncharacterized protein ZK112.3 OS=Caenorhabditis elegans GN=ZK112.3 PE=4 SV=1	0.04	Putative proline-rich protein 21 (Fragment) OS=Hydra vulgaris GN=PRR21 PE=2 SV=1	9.00E-06	hypothetical protein [Spirulina subsalsa]	1.00E-04
isogroup19349	845	55	11	2.04	9.64E-07	Baculoviral IAP repeat-containing protein 7-A OS=Xenopus laevis GN=birc7-a PE=1 SV=1	1.00E-07	Uncharacterized protein OS=Capitella teleta GN=CAPTEDRAFT_120337 PE=4 SV=1	8.00E-08	PREDICTED: baculoviral IAP repeat-containing protein 4-like [Saccoglossus kowalevskii]	2.00E-08
isogroup21510	723	47	10	1.95	1.09E-05	Protein ZBED8 OS=Bos taurus GN=ZBED8 PE=2 SV=1	9.00E-15	Chromosome undetermined SCAF7481, whole genome shotgun sequence. (Fragment) OS=Tetraodon nigroviridis GN=GSTENG00004516001 PE=4 SV=1	9.00E-18	unnamed protein product [Tetraodon nigroviridis]	6.00E-18
isogroup09861	2579	335	75	1.88	0	Fibroblast growth factor receptor-like 1 OS=Mus musculus GN=Fgfrl1 PE=1 SV=1	2.00E-18	NOU-DARAKE OS=Dugesia japonica GN=nou-darake PE=2 SV=1	0	NOU-DARAKE [Dugesia japonica]	0
isogroup13916	1323	56	13	1.82	4.41E-06	Zinc finger protein ZIC 1 OS=Gallus gallus GN=ZIC1 PE=1 SV=1	1.00E-91	Zinc finger protein Dj-ZicA OS=Dugesia japonica GN=Dj-ZicA PE=2 SV=1	0	zinc finger protein Dj-ZicA [Dugesia japonica]	0
isogroup25913	200	72	17	1.80	2.49E-07	Synaptic vesicle glycoprotein 2B OS=Mus musculus GN=Sv2b PE=1 SV=1	7.2	Predicted protein (Fragment) OS=Nematostella vectensis GN=v1g74567 PE=4 SV=1	4.00E-13	predicted protein [Nematostella vectensis] gb|EDO36545.1| predicted protein [Nematostella vectensis]	3.00E-13
isogroup04716	9110	1775	424	1.78	0	Translation initiation factor IF-2 OS=Persephonella marina (strain DSM 14350 / EX-H1) GN=infB PE=3 SV=1	0.089	Predicted protein OS=Physcomitrella patens subsp. patens GN=PHYPADRAFT_66556 PE=4 SV=1	8.00E-10	predicted protein [Physcomitrella patens subsp. patens] gb|EDQ81290.1| predicted protein [Physcomitrella patens subsp. patens]	6.00E-10
contig16471	943	50	12	1.77	2.05E-05	Retrovirus-related Pol polyprotein from transposon 297 OS=Drosophila melanogaster GN=pol PE=4 SV=1	7.00E-24	Retrovirus polyprotein OS=Dicentrarchus labrax GN=DLA_It03860 PE=4 SV=1	1.00E-24	PREDICTED: uncharacterized protein LOC101886116 isoform X1 [Danio rerio] ref|XP_005172039.1| PREDICTED: uncharacterized protein LOC101886116 isoform X2 [Danio rerio] ref|XP_005172040.1| PREDICTED: uncharacterized protein LOC101886116 isoform X3 [Danio rerio] ref|XP_005172041.1| PREDICTED: uncharacterized protein LOC101886116 isoform X4 [Danio rerio]	7.00E-26
isogroup24538	490	173	42	1.76	3.44E-15	Apolipophorins OS=Drosophila melanogaster GN=Rfabg PE=1 SV=2	5.00E-14	Uncharacterized protein OS=Tupaia chinensis GN=TREES_T100018447 PE=4 SV=1	1.00E-21	PREDICTED: vitellogenin-like, partial [Pongo abelii]	2.00E-22
isogroup05528	1764	41	10	1.75	0.000132148	Probable DNA polymerase OS=Podospora anserina PE=3 SV=1	0.073	Protein CBG15088 OS=Caenorhabditis briggsae GN=CBG15088 PE=4 SV=2	2.00E-25	Protein CBG25579 [Caenorhabditis briggsae]	3.00E-32
isogroup03889	443	73	18	1.74	4.01E-07	PR domain zinc finger protein 12 OS=Mus musculus GN=Prdm12 PE=3 SV=1	2.00E-06	Predicted protein (Fragment) OS=Nematostella vectensis GN=v1g119550 PE=4 SV=1	1.00E-07	predicted protein [Nematostella vectensis] gb|EDO36495.1| predicted protein [Nematostella vectensis]	7.00E-08
contig13483	934	259	65	1.71	0	Zinc finger protein 737 OS=Homo sapiens GN=ZNF737 PE=2 SV=3	0.22	Collagen alpha 1(VII) chain OS=Echinococcus multilocularis GN=EmuJ_000619300 PE=4	4.00E-10	collagen alpha 1VII chain [Echinococcus granulosus]	2.00E-09
isogroup06194	1447	222	57	1.68	0	Protein Wnt-2b-A OS=Xenopus laevis GN=wnt2b-a PE=2 SV=1	7.00E-96	Protein Wnt (Fragment) OS=Dugesia japonica GN=DjwntB PE=2 SV=1	0	WNT [Dugesia japonica]	0
isogroup05208	2136	151	40	1.63	2.92E-12	Frizzled-8 OS=Xenopus laevis GN=fzd8 PE=1 SV=1	1.00E-109	Frizzled receptor OS=Dugesia japonica GN=DjfzA PE=2 SV=1	0	frizzled receptor [Dugesia japonica]	0

isogroup17454	981	108	29	1.61	4.81E-09	Secreted frizzled-related protein 2 OS=Mus musculus GN=Sfrp2 PE=2 SV=2	4.00E-45	Secreted frizzled-related protein A (Fragment) OS=Dugesia japonica GN=DjsFRP-A PE=2	0	secreted frizzled-related protein A [Dugesia japonica]	0
isogroup09578	2854	1008	271	1.61	0	Uncharacterized protein ORF91 OS=Phalaenopsis aphrodite subsp. formosana PE=4 SV=1	8.00E-14	Putative uncharacterized protein OS=Clostridium carboxidivorans P7 GN=CLCAR_4307 PE=4 SV=1	8.00E-62	hypothetical protein [Clostridium carboxidivorans] gb|EFG86088.1| hypothetical protein CLCAR_4307 [Clostridium carboxidivorans P7]	6.00E-62
isogroup19020	870	37	10	1.60	0.000644894	Ras-related protein Rap-1 OS=Caenorhabditis elegans GN=rap-1 PE=3 SV=1	2.00E-25	Uncharacterized protein OS=Lottia gigantea GN=LOTGIDRAFT_229516 PE=4 SV=1	1.00E-31	hypothetical protein LOTGIDRAFT_229516 [Lottia gigantea]	9.00E-32
isogroup00813	14144	60001	16218	1.60	0	Replicase polyprotein OS=Cricket paralysis virus (isolate Teleogryllus commodus/Australia/CrPVVIC/1968) GN=ORF1 PE=1 SV=1	2.00E-45	Non-structural polyprotein OS=Formica exsecta virus 1 GN=FEX1gp1 PE=4 SV=1	3.00E-49	nonstructural polyprotein [Solenopsis invicta virus-1] gb|AAU85375.1| nonstructural polyprotein [Solenopsis invicta virus-1]	2.00E-46
contig20555	3806	6709	1817	1.60	0	Apolipophorins OS=Locusta migratoria PE=1 SV=2	2.00E-35	Uncharacterized protein OS=Crassostrea gigas GN=CGI_10002658 PE=4 SV=1	1.00E-84	hypothetical protein CGI_10002658 [Crassostrea gigas]	7.00E-85
contig13535	611	55	15	1.59	3.54E-05	Zonadhesin OS=Mus musculus GN=Zan PE=2 SV=1	8.00E-12	Putative von Willebrand factor type A domain-containing protein OS=Schistosoma mansoni GN=Smp_157690 PE=4 SV=1	5.00E-18	putative von Willebrand factor type A domain-containing protein [Schistosoma mansoni]	3.00E-18
isogroup13604	1377	66	18	1.59	5.89E-06	Forkhead box protein J1.2 OS=Xenopus laevis GN=foxj1.2 PE=2 SV=1	1.00E-34	Forkhead box J1-like protein 1 OS=Schmidtea mediterranea PE=2 SV=1	1.00E-169	forkhead box J1-like protein 1 [Schmidtea mediterranea]	1.00E-169
isogroup23274	607	2439	666	1.59	0	Protein E7 OS=Human papillomavirus type 55 GN=E7 PE=3 SV=1	0.59	Uncharacterized protein (Fragment) OS=Schmidtea mediterranea PE=2 SV=1	8.00E-23	hypothetical protein, partial [Schmidtea mediterranea]	5.00E-23
contig20552	1786	1575	435	1.57	0	Protein ycf2 OS=Marchantia polymorpha GN=ycf2 PE=3 SV=1	2.00E-05	Uncharacterized protein OS=Fusobacterium periodonticum ATCC 33693 GN=FUSPEROL_02206 PE=4 SV=1	9.00E-07	hypothetical protein [Fusobacterium periodonticum] gb|EFE85848.1| hypothetical protein FUSPEROL_02206 [Fusobacterium periodonticum ATCC 33693]	6.00E-07
isogroup07395	946	36	10	1.56	0.000947792	Cellular nucleic acid-binding protein homolog OS=Schizosaccharomyces pombe (strain 972 / ATCC 24843) GN=byr3 PE=4 SV=1	2.00E-11	Singapore isolate B (sub-type 7) whole genome shotgun sequence assembly, scaffold_21 OS=Blastocystis hominis GN=GSBLH_T00002894001 PE=4 SV=1	1.00E-11	PREDICTED: CCHC-type zinc finger protein CG3800-like isoform 1 [Bombus terrestris] ref|XP_003397960.1| PREDICTED: CCHC-type zinc finger protein CG3800-like isoform 2 [Bombus terrestris] ref|XP_003485390.1| PREDICTED: CCHC-type zinc finger protein CG3800-like	1.00E-12
contig20548	1479	6341	1811	1.52	0	Apolipophorins OS=Locusta migratoria PE=1 SV=2	2.00E-13	Apolipophorin OS=Ascaris suum GN=ASU_02895 PE=4 SV=1	6.00E-27	PREDICTED: zonadhesin-like [Saccoglossus kowalevskii]	4.00E-30
isogroup16347	1073	49	14	1.52	0.000154728	Unconventional myosin-Va OS=Mus musculus GN=Myo5a PE=1 SV=2	2.00E-07	NAD-dependent DNA ligase OS=Pleurocapsa sp. PCC 7327 GN=Ple7327_4433 PE=4 SV=1	1.00E-07	NAD-dependent DNA ligase [Pleurocapsa sp. PCC 7327] ref|WP_015145834.1| NAD-dependent DNA ligase [Pleurocapsa minor] gb|AFY79538.1| NAD-dependent DNA	8.00E-08
isogroup21173	744	542	155	1.52	0	Elongation factor 4 OS=Leptospira borgpetersenii serovar Hardjo-bovis (strain L550) GN=lepA PE=3 SV=1	0.011	SJCHGC05825 protein (Fragment) OS=Schistosoma japonicum PE=2 SV=2	7.00E-37	SJCHGC05825 protein [Schistosoma japonicum]	5.00E-37
isogroup14546	1254	150	43	1.52	4.00E-11	Silk gland factor 1 OS=Bombyx mori GN=SGF1 PE=2 SV=1	1.00E-68	Winged helix/forkhead transcription factor DjFoxA OS=Dugesia japonica GN=DjFoxA	0	winged helix/forkhead transcription factor DjFoxA [Dugesia japonica]	0
isogroup10556	2109	225	65	1.51	8.88E-16	Zinc finger protein ZIC 2 OS=Homo sapiens GN=ZIC2 PE=1 SV=2	7.00E-84	Zinc finger protein Dj-ZicB OS=Dugesia japonica GN=Dj-ZicB PE=2 SV=1	0	zinc finger protein Dj-ZicB [Dugesia japonica]	0
isogroup23806	564	38	11	1.50	0.000961778	Dynein heavy chain (Fragment) OS=Oncorhynchus mykiss PE=2 SV=1	0.01	Uncharacterized protein OS=Helobdella robusta GN=HELRODRAFT_176662 PE=4 SV=1	4.00E-10	hypothetical protein HELRODRAFT_176662 [Helobdella robusta]	2.00E-10
contig13534	690	55	16	1.50	7.56E-05	Kielin/chordin-like protein OS=Mus musculus GN=Kcp PE=1 SV=2	8.00E-10	IgGFc-binding protein OS=Crassostrea gigas GN=CGI_10015609 PE=4 SV=1	2.00E-33	IgGFc-binding protein [Crassostrea gigas]	1.00E-33
isogroup10908	1952	41	12	1.49	0.000667435	Putative ankyrin repeat protein L66 OS=Acanthamoeba polyphaga mimivirus GN=MIMI_L66 PE=4 SV=1	0.01	Location of vulva defective 1 OS=Crassostrea gigas GN=CGI_10007022 PE=4 SV=1	2.00E-24	Location of vulva defective 1 [Crassostrea gigas]	2.00E-24
isogroup18798	885	41	12	1.49	0.000667435	Homeobox protein SIX6 OS=Gallus gallus GN=SIX6 PE=2 SV=1	4.00E-86	Six3-1 (Fragment) OS=Schmidtea mediterranea PE=2 SV=1	1.00E-123	six3-1, partial [Schmidtea mediterranea]	1.00E-124
isogroup08547	309	112	33	1.48	2.18E-08	Protein PF14_0175 OS=Plasmodium falciparum (isolate 3D7) GN=PF14_0175 PE=4 SV=1	1.1	Putative uncharacterized protein (Fragment) OS=Camponotus floridanus GN=EAG_03031 PE=4 SV=1	2.00E-12	hypothetical protein EAG_03031 [Camponotus floridanus]	2.00E-12
contig13499	1171	207	61	1.48	2.78E-14	Probable DNA repair protein RAD50 OS=Plasmodium falciparum (isolate 3D7) GN=PFF0285c PE=3 SV=1	3.5	Putative von Willebrand factor type A domain-containing protein OS=Schistosoma mansoni GN=Smp_157690 PE=4 SV=1	9.00E-06	putative von Willebrand factor type A domain-containing protein [Schistosoma mansoni]	6.00E-06
isogroup22411	673	79	24	1.43	4.31E-06			Uncharacterized protein (Fragment) OS=Lottia gigantea GN=LOTGIDRAFT_148500 PE=4	2.00E-12	hypothetical protein LOTGIDRAFT_148500, partial [Lottia gigantea]	1.00E-12
isogroup03845	1089	492	150	1.43	0	Signal recognition particle receptor FtsY OS=Sulfolobus acidocaldarius (strain ATCC 33909 / DSM 639 / JCM 8929 / NBRC 15157 / NCIMB 11770) GN=ftsY PE=3 SV=1	7.2	Putative uncharacterized protein OS=Daphnia pulex GN=DAPPUDRAFT_317650 PE=4 SV=1	6.00E-15	hypothetical protein DAPPUDRAFT_317650 [Daphnia pulex]	5.00E-15
isogroup01942	433	308	94	1.43	0	Epsin OS=Dictyostelium discoideum GN=epnA PE=3 SV=1	2.5	Uncharacterized protein (Fragment) OS=Ficedula albicollis PE=4 SV=1	7.00E-09	PREDICTED: BAT2 domain containing 1-like [Saccoglossus kowalevskii]	7.00E-09
isogroup14946	1208	72	22	1.43	1.25E-05	SH3 domain-binding protein 4-A OS=Xenopus laevis GN=sh3bp4-a PE=2 SV=1	0.76	Uncharacterized protein OS=Crassostrea gigas GN=CGI_10008774 PE=4 SV=1	1.00E-15	PREDICTED: uncharacterized protein LOC101848372 isoform X1 [Aplysia californica] ref|XP_005104171.1| PREDICTED: uncharacterized protein LOC101848372 isoform X2 [Aplysia californica]	3.00E-17
isogroup15224	1169	62	19	1.42	5.23E-05	Uncharacterized protein C1751.04 OS=Schizosaccharomyces pombe (strain 972 / ATCC 24843) GN=SPAC1751.04 PE=4 SV=2	6.9	Uncharacterized protein OS=Acyrthosiphon pisum PE=4 SV=1	1.00E-21	conserved hypothetical protein [Trichinella spiralis] gb|EFV50790.1| conserved hypothetical protein [Trichinella spiralis]	1.00E-21

isogroup19067	867	68	21	1.41	2.54E-05	Replicase polyprotein 1ab OS=Porcine transmissible gastroenteritis coronavirus (strain Purdue) GN=rep PE=1 SV=1	1.7	Putative uncharacterized protein (Fragment) OS=Acromyrmex echinatior GN=G5I_03377 PE=4 SV=1	3.00E-12	hypothetical protein G5I_03377 [Acromyrmex echinatior]	2.00E-12
isogroup12727	1513	48	15	1.39	0.000454846	Mariner Mos1 transposase OS=Drosophila mauritiana GN=mariner\T PE=1 SV=1	8.00E-04	Uncharacterized protein (Fragment) OS=Bos mutus GN=M91_17600 PE=4 SV=1	8.00E-14	PREDICTED: histone-lysine N-methyltransferase SETMAR-like [Bos taurus] ref|XP_005218920.1| PREDICTED: histone-lysine N-methyltransferase SETMAR-	2.00E-15
isogroup22459	672	48	15	1.39	0.000454846	cAMP-dependent protein kinase type I-beta regulatory subunit OS=Homo sapiens GN=PRKAR1B PE=1 SV=4	3.00E-10	Uncharacterized protein OS=Lottia gigantea GN=LOTGIDRAFT_227784 PE=4 SV=1	7.00E-36	hypothetical protein LOTGIDRAFT_227784 [Lottia gigantea]	5.00E-36
isogroup12706	1514	54	17	1.38	0.000217931	Zinc finger C2HC domain-containing protein 1A OS=Xenopus laevis GN=zc2hc1a PE=2 SV=1	3.00E-10	Uncharacterized protein OS=Amphimedon queenslandica GN=LOC100634074 PE=4 SV=1	3.00E-12	PREDICTED: hypothetical protein LOC100634074 [Amphimedon queenslandica]	2.00E-12
isogroup15935	1113	54	17	1.38	0.000217931	Protein tailless OS=Drosophila virilis GN=tll PE=3 SV=1	6.00E-51	Nuclear receptor TLX-1 OS=Schmidtea mediterranea GN=tlx-1 PE=2 SV=1	1.00E-176	nuclear receptor TLX-1 [Schmidtea mediterranea]	1.00E-176
contig13523	663	163	52	1.36	2.07E-10	Bowman-Birk type trypsin inhibitor OS=Hordeum vulgare GN=BBBI PE=1 SV=1	0.54	Collagen alpha 1VII chain OS=Echinococcus granulosus GN=EgrG_000619300 PE=4 SV=1	2.00E-08	collagen alpha 1VII chain [Echinococcus granulosus]	2.00E-08
isogroup18347	880	47	15	1.36	0.000644795	DNA polymerase OS=Acanthamoeba polyphaga mimivirus GN=POLB PE=3 SV=1	1.00E-21	DNA polymerase (Fragment) OS=Megavirus courdo5 PE=3 SV=1	2.00E-23	B-family DNA polymerase, partial [Mimivirus Bus] gb|AFM52364.1| B-family DNA polymerase, partial [Megavirus courdo5]	2.00E-23
isogroup25915	201	175	56	1.36	5.04E-11	Uncharacterized protein C2orf16 OS=Homo sapiens GN=C2orf16 PE=2 SV=3	1.00E-14	Melatonin-related receptor OS=Pteropus alecto GN=PAL_GLEAN10000992 PE=3 SV=1	2.00E-15	PREDICTED: uncharacterized protein LOC101885061 [Danio rerio]	1.00E-20
isogroup08766	610	134	43	1.36	9.69E-09	Cuticle protein 16.5, isoform A OS=Locusta migratoria PE=1 SV=1	3.3	Uncharacterized protein OS=Lottia gigantea GN=LOTGIDRAFT_145636 PE=4 SV=1	4.00E-10	hypothetical protein LOTGIDRAFT_145636 [Lottia gigantea]	2.00E-10
isogroup10846	1975	124	40	1.35	3.92E-08	Frizzled-5 OS=Xenopus laevis GN=fzd5 PE=2 SV=1	0	Fzd-5/8-1 OS=Schmidtea mediterranea PE=2 SV=1	0	PREDICTED: LOW QUALITY PROTEIN: frizzled-5-like [Aplysia californica]	0
isogroup25830	221	96	31	1.35	1.36E-06	Delta(24(24(1)))-sterol reductase OS=Saccharomyces cerevisiae (strain ATCC 204508 / S288c) GN=ERG4 PE=1 SV=2	0.017	Uncharacterized protein (Fragment) OS=Helobdella robusta GN=HELRODRAFT_90243 PE=4 SV=1	3.00E-09	hypothetical protein HELRODRAFT_90243, partial [Helobdella robusta]	2.00E-09
isogroup12449	1559	609	200	1.32	0	Fibroblast growth factor receptor-like 1 OS=Gallus gallus GN=FGFRL1 PE=1 SV=1	1.00E-11	Smed-NDK-3 (Fragment) OS=Schmidtea mediterranea PE=2 SV=1	1.00E-179	Smed-NDK-3 [Schmidtea mediterranea]	1.00E-180
isogroup13228	1432	76	25	1.32	2.31E-05	Procollagen-lysine,2-oxoglutarate 5-dioxygenase 2 OS=Mus musculus GN=Plod2 PE=2 SV=2	1.00E-132	Procollagen-lysine, 2-oxoglutarate 5-dioxygenase 3 OS=Schistosoma japonicum GN=plod-prov PE=2 SV=1	1.00E-148	procollagen-lysine, 2-oxoglutarate 5-dioxygenase 3 [Schistosoma japonicum]	1.00E-149
isogroup13050	1462	469	156	1.30	0	Minor capsid protein L2 OS=Human papillomavirus type 45 GN=L2 PE=3 SV=1	2.2	Uncharacterized protein OS=Collinsella aerofaciens ATCC 25986 GN=COLAER_01671 PE=4 SV=1	5.00E-36	hypothetical protein [Collinsella aerofaciens] gb|EBA38630.1| hypothetical protein COLAER_02284 [Collinsella aerofaciens ATCC 25986] gb|EBA38843.1| hypothetical protein COLAER_01925 [Collinsella aerofaciens ATCC 25986] gb|EBA39061.1| hypothetical protein COLAER_01671 [Collinsella aerofaciens ATCC 25986]	4.00E-36
isogroup12940	1473	105	35	1.30	8.67E-07	Zinc finger protein GIS2 OS=Saccharomyces cerevisiae (strain ATCC 204508 / S288c) GN=GIS2 PE=1 SV=1	4.00E-06	Piso0_005509 protein OS=Pichia sorbitophila (strain ATCC MYA-4447 / BCRC 22081 / CBS 7064 / NBRC 10061 / NRRL Y-12695) GN=Piso0_005509 PE=4 SV=1	1.00E-06	Piso0_005509 [Millerozyma farinosa CBS 7064] emb|CCE85873.1| Piso0_005509 [Millerozyma farinosa CBS 7064]	9.00E-07
isogroup16323	1070	48	16	1.30	0.000880055	Neuronal acetylcholine receptor subunit alpha-10 OS=Gallus gallus GN=CHRNA10 PE=3	1.00E-91	Uncharacterized protein (Fragment) OS=Lottia gigantea GN=LOTGIDRAFT_102597 PE=3	1.00E-152	hypothetical protein LOTGIDRAFT_102597, partial [Lottia gigantea]	1.00E-152
isogroup00275	6071	12952	4336	1.29	0	SCO-spondin OS=Gallus gallus GN=SSPO PE=2 SV=1	7.00E-18	Uncharacterized protein (Fragment) OS=Latimeria chalumnae PE=4 SV=1	4.00E-17	PREDICTED: SCO-spondin-like [Latimeria chalumnae]	3.00E-17
isogroup22285	682	3349	1132	1.28	0	Soma ferritin OS=Lymnaea stagnalis PE=2 SV=2	4.00E-71	Ferritin (Fragment) OS=Branchiostoma lanceolatum PE=2 SV=2	8.00E-76	ferritin heavy chain polypeptide 1 [Branchiostoma lanceolatum]	6.00E-76
contig12785	1400	65	22	1.28	0.000133376	Histone-lysine N-methyltransferase SETMAR OS=Homo sapiens GN=SETMAR PE=1 SV=1	2.00E-11	Histone-lysine N-methyltransferase SETMAR (Fragment) OS=Bos mutus GN=M91_04391 PE=4 SV=1	3.00E-12	PREDICTED: histone-lysine N-methyltransferase SETMAR-like [Pantholops hodgsonii]	3.00E-13
isogroup14901	1212	96	33	1.26	4.66E-06	WD repeat-containing protein 86 OS=Homo sapiens GN=WDR86 PE=2 SV=3	1.00E-83	Uncharacterized protein OS=Capitella teleta GN=CAPTEDRAFT_115707 PE=4 SV=1	1.00E-123	hypothetical protein CAPTEDRAFT_115707 [Capitella teleta]	1.00E-124
isogroup04007	3129	654	225	1.26	0	Protein ycf2 OS=Physcomitrella patens subsp. patens GN=ycf2 PE=3 SV=1	2.00E-06	Putative phenylalanyl-tRNA synthetase, beta subunit OS=Zinderia insecticola (strain CARI) GN=pheT PE=3 SV=1	1.00E-08	putative phenylalanyl-tRNA synthetase subunit beta [Candidatus Zinderia insecticola CARI] ref|WP_013315231.1| putative phenylalanyl-tRNA synthetase subunit beta [Candidatus Zinderia insecticola] gb|ADM89654.1| putative phenylalanyl-tRNA synthetase, beta subunit	8.00E-09
isogroup08369	420	58	20	1.25	0.00038578	Kyphoscoliosis peptidase OS=Homo sapiens GN=KY PE=1 SV=2	5.00E-16	SJCHGC08483 protein (Fragment) OS=Schistosoma japonicum PE=2 SV=2	1.00E-55	SJCHGC08483 protein [Schistosoma japonicum]	1.00E-55
isogroup18026	934	55	19	1.25	0.000558565	Chromosomal replication initiator protein DnaA OS=Bifidobacterium longum subsp. infantis (strain ATCC 15697 / DSM 20088 / JCM 1222 / NCTC 11817 / S12) GN=dnaA PE=3 SV=1	8.4	Putative uncharacterized protein OS=Tribolium castaneum GN=TcasGA2_TC009398 PE=4 SV=1	2.00E-33	hypothetical protein TcasGA2_TC009398 [Tribolium castaneum]	1.00E-33
isogroup07545	870	891	310	1.24	0	PLAC8-like protein 1 OS=Homo sapiens GN=PLAC8L1 PE=2 SV=1	6.00E-16	Placenta-specific gene 8 protein OS=Schistosoma japonicum PE=4 SV=1	4.00E-26	Placenta-specific gene 8 protein [Schistosoma japonicum]	3.00E-26
contig15998	1432	60	21	1.23	0.000368143	Histone-lysine N-methyltransferase SETMAR OS=Homo sapiens GN=SETMAR PE=1 SV=1	3.00E-32	Histone-lysine N-methyltransferase OS=Homo sapiens GN=SETMAR PE=2 SV=1	2.00E-30	mariner transposase [Homo sapiens]	1.00E-31
isogroup01861	1233	593	208	1.23	0	Solute carrier family 43 member 3 OS=Homo sapiens GN=SLC43A3 PE=1 SV=2	7.00E-11	Solute carrier family 43 OS=Hymenolepis microstoma GN=HmN_000456700 PE=4 SV=1	3.00E-14	solute carrier family 43 [Hymenolepis microstoma]	2.00E-14

isogroup09937	2510	88	31	1.22	1.80E-05	Muscle M-line assembly protein unc-89 OS=Caenorhabditis elegans GN=unc-89 PE=1 SV=3	9.00E-24	Titin OS=Echinococcus multilocularis GN=EmuJ_000610500 PE=4 SV=1	2.00E-29	titin [Clonorchis sinensis]	7.00E-28
isogroup13092	1450	56	20	1.20	0.000732596	FMRFamide-activated amiloride-sensitive sodium channel OS=Helix aspersa PE=2 SV=1	5.00E-09	FMRFamide activated amiloride sensitive sodium OS=Echinococcus multilocularis GN=EmuJ_000738300 PE=3 SV=1	5.00E-79	FMRFamide activated amiloride sensitive sodium [Echinococcus granulosus]	2.00E-78
isogroup09448	3033	308	111	1.19	4.11E-15	Rho GTPase-activating protein 100F OS=Drosophila melanogaster GN=RhoGAP100F PE=1 SV=2	9.00E-76	Rho GTPase activating protein OS=Echinococcus multilocularis GN=EmuJ_000441500 PE=4 SV=1	1.00E-103	rho GTPase activating protein [Echinococcus granulosus]	1.00E-102
contig00128	1203	86	31	1.19	3.35E-05	Hepatocyte nuclear factor 6 OS=Rattus norvegicus GN=Onecut1 PE=1 SV=1	4.00E-44	Uncharacterized protein (Fragment) OS=Loxodonta africana GN=LOC100676371 PE=3 SV=1	8.00E-47	PREDICTED: LOW QUALITY PROTEIN: hepatocyte nuclear factor 6-like [Loxodonta africana]	6.00E-46
isogroup10011	2462	465	170	1.17	0	Fibroblast growth factor receptor-like 1 OS=Gallus gallus GN=FGFRL1 PE=1 SV=1	2.00E-14	NDL-4 OS=Dendrocoelum lacteum PE=2 SV=1	9.00E-79	Smed-NDK-4 [Schmidtea mediterranea]	5.00E-77
isogroup14430	1265	93	34	1.17	2.09E-05	Uncharacterized MFS-type transporter YhjX OS=Escherichia coli (strain K12) GN=yhjX PE=1 SV=1	2.00E-27	Putative uncharacterized protein OS=Branchiostoma floridae GN=BRAFLDRAFT_120194 PE=4 SV=1	1.00E-103	hypothetical protein BRAFLDRAFT_120194 [Branchiostoma floridae] gb|EEN49081.1| hypothetical protein BRAFLDRAFT_120194 [Branchiostoma floridae]	1.00E-103
isogroup06039	1411	82	30	1.17	6.53E-05	Beclin-1 OS=Xenopus tropicalis GN=becn1 PE=2 SV=1	2.00E-05	Predicted protein OS=Naegleria gruberi GN=NAEGRDRAFT_79948 PE=4 SV=1	5.00E-06	PREDICTED: trichohyalin [Ochotona princeps]	2.00E-06
isogroup09762	2669	171	63	1.16	1.03E-08	Histone-lysine N-methyltransferase SETMAR OS=Homo sapiens GN=SETMAR PE=1 SV=1	3.00E-09	Transposase OS=Forficula auricularia PE=4 SV=1	3.00E-28	PREDICTED: histone-lysine N-methyltransferase SETMAR-like [Apis florea]	7.00E-31
contig13515	1122	473	175	1.15	0	Ribosomal operon-associated A protein OS=Euglena gracilis GN=roaA PE=3 SV=1	2.00E-04	Collagen alpha-1(VII) chain (Fragment) OS=Clonorchis sinensis GN=CLF_109355 PE=4 SV=1	3.00E-08	collagen alpha-1(VII) chain, partial [Clonorchis sinensis]	2.00E-08
isogroup11570	1752	86	32	1.14	5.80E-05	Histone-lysine N-methyltransferase SETMAR OS=Homo sapiens GN=SETMAR PE=1 SV=1	8.00E-63	cDNA FLJ54643, highly similar to Homo sapiens SET domain and mariner transposase fusion gene (SETMAR), mRNA OS=Homo	4.00E-63	transposase [synthetic construct]	2.00E-68
isogroup07377	754	142	53	1.14	2.59E-07	Pentatricopeptide repeat-containing protein 2 mitochondrial OS=Xenopus laevis GN=ptcd2 PE=2 SV=1	1.8	Uncharacterized protein (Fragment) OS=Nasonia vitripennis PE=4 SV=1	5.00E-10	hypothetical protein G5I_03377 [Acromyrmex echinatior]	1.00E-04
contig17607	1866	563	212	1.13	0	Plasminogen OS=Rattus norvegicus GN=Plg PE=2 SV=2	3.00E-04	Uncharacterized protein OS=Helobdella robusta GN=HELRODRAFT_166364 PE=4 SV=1	1.00E-06	hypothetical protein HELRODRAFT_166364 [Helobdella robusta]	1.00E-06
isogroup17647	966	111	42	1.12	7.10E-06	Gag polyprotein OS=Jembrana disease virus GN=gag PE=3 SV=1	4.00E-07	Similar to Cellular nucleic acid-binding protein homolog acc. no. P36627 OS=Pyronema omphalodes (strain CBS 100304) GN=PCON_07655 PE=4 SV=1	3.00E-07	Similar to Cellular nucleic acid-binding protein homolog; acc. no. P36627 [Pyronema omphalodes CBS 100304]	2.00E-07
isogroup02063	1160	155	59	1.11	1.34E-07	Importin subunit alpha-3 OS=Caenorhabditis elegans GN=ima-3 PE=1 SV=2	6.00E-09	Karyopherin alpha 4 (Fragment) OS=Ichthyophis bannanicus PE=2 SV=1	5.00E-09	karyopherin alpha 4 [Ichthyophis bannanicus]	3.00E-09
isogroup16938	1019	76	29	1.11	0.000230042	Aminomethyltransferase OS=Bacillus licheniformis (strain DSM 13 / ATCC 14580) GN=gcvT PE=3 SV=1	0.51	Uncharacterized protein OS=Lottia gigantea GN=LOTGIDRAFT_155939 PE=4 SV=1	2.00E-12	hypothetical protein LOTGIDRAFT_155939 [Lottia gigantea]	1.00E-12
isogroup17540	976	76	29	1.11	0.000230042	ADP,ATP carrier protein 3 OS=Saccharomyces cerevisiae (strain ATCC 204508 / S288c) GN=AAC3 PE=1 SV=1	1.00E-122	Uncharacterized protein OS=Capitella teleta GN=CAPTEDRAFT_163594 PE=3 SV=1	1.00E-162	hypothetical protein CAPTEDRAFT_163594 [Capitella teleta]	1.00E-162
isogroup10042	2445	466	178	1.10	0	Frizzled-5 OS=Xenopus laevis GN=fzd5 PE=2 SV=1	0	Fzd-5/8-4 OS=Schmidtea mediterranea PE=2 SV=1	0	hypothetical protein LOTGIDRAFT_200873 partial [Lottia gigantea]	0
isogroup25838	218	94	36	1.10	4.49E-05	Putative uncharacterized protein DDB_G0291608 OS=Dictyostelium discoideum GN=DDB_G0291608 PE=4 SV=1	0.005	Mucin OS=Schistosoma mansoni PE=2 SV=1	3.00E-07	mucin [Schistosoma mansoni]	2.00E-07
isogroup16007	1104	104	40	1.09	1.93E-05	Cellular nucleic acid-binding protein homolog OS=Schizosaccharomyces pombe (strain 972 / ATCC 24843) GN=byr3 PE=4 SV=1	2.00E-11	Uncharacterized protein (Fragment) OS=Bos taurus GN=ZCCHC13 PE=4 SV=1	1.00E-11	PREDICTED: CCHC-type zinc finger protein CG3800-like isoform 1 [Bombus terrestris] ref|XP_003397960.1| PREDICTED: CCHC-type zinc finger protein CG3800-like isoform 2 [Bombus terrestris] ref|XP_003485390.1| PREDICTED: CCHC-type zinc finger protein CG3800-like	2.00E-12
isogroup06833	1435	96	37	1.09	4.20E-05	Putative ankyrin repeat protein L25 OS=Acanthamoeba polyphaga mimivirus GN=MIMI_L25 PE=4 SV=1	5.00E-17	AAEL014742-PA (Fragment) OS=Aedes aegypti GN=AAEL014742 PE=4 SV=1	1.00E-36	ankyrin 2,3/unc44 [Aedes aegypti] gb|EAT33004.1| AAEL014742-PA, partial [Aedes aegypti]	1.00E-36
isogroup01785	2385	88	34	1.09	9.16E-05	Pre-mRNA-splicing factor CWC22 OS=Debaryomyces hansenii (strain ATCC 36239 / CBS 767 / JCM 1990 / NBRC 0083 / IGC 2968) GN=CWC22 PE=3 SV=2	0.16	Transposase (Fragment) OS=Stylochus zebra PE=4 SV=1	2.00E-11	PREDICTED: uncharacterized protein LOC100899128 [Metaseiulus occidentalis]	1.00E-15
isogroup25306	331	88	34	1.09	9.16E-05	Soluble scavenger receptor cysteine-rich domain-containing protein SSC5D OS=Homo sapiens GN=SSC5D PE=2 SV=3	8.00E-09	WGS project CAEQ00000000 data, annotated contig 2455 OS=Trypanosoma congolense (strain IL3000) GN=TCIL3000_0_61090 PE=4	6.00E-35	unnamed protein product [Trypanosoma congolense IL3000]	4.00E-35
contig13471	1840	581	227	1.07	0	Ribosomal operon-associated A protein OS=Euglena gracilis GN=roaA PE=3 SV=1	0.012	Collagen alpha-1(VII) chain (Fragment) OS=Clonorchis sinensis GN=CLF_109355 PE=4 SV=1	2.00E-12	collagen alpha-1(VII) chain, partial [Clonorchis sinensis]	1.00E-12
isogroup09295	3279	110	43	1.07	1.57E-05	ATP-binding cassette sub-family A member 1 OS=Mus musculus GN=Abca1 PE=1 SV=4	1.00E-178	Uncharacterized protein OS=Lottia gigantea GN=LOTGIDRAFT_212836 PE=4 SV=1	0	PREDICTED: ATP-binding cassette sub-family A member 1-like [Aplysia californica]	0
isogroup01857	984	69	27	1.07	0.000634201	Zinc finger protein GIS2 OS=Saccharomyces cerevisiae (strain ATCC 204508 / S288c) GN=GIS2 PE=1 SV=1	3.00E-09	Probable Zinc finger protein GIS2 OS=Zygosaccharomyces bailii ISA1307 GN=ZbGIS2 PE=4 SV=1	4.00E-08	hypothetical protein FPSE_03593 [Fusarium pseudograminearum CS3096]	6.00E-07

isogroup06033	1422	99	39	1.06	4.86E-05	tRNA (guanine(37)-N1)-methyltransferase OS=Plasmodium falciparum (isolate 3D7) GN=PFI0700c PE=3 SV=1	1.8	Transposable element Tc3 transposase (Fragment) OS=Camponotus floridanus GN=EAG_14192 PE=4 SV=1	9.00E-21	Transposable element Tc3 transposase [Camponotus floridanus]	6.00E-21
isogroup05272	2235	241	95	1.06	2.39E-10	E3 ubiquitin-protein ligase XIAP OS=Xenopus tropicalis GN=xiap PE=2 SV=1	5.00E-22	E3 ubiquitin-protein ligase XIAP OS=Xenopus tropicalis GN=xiap PE=4 SV=1	1.00E-20	PREDICTED: E3 ubiquitin-protein ligase XIAP [Alligator sinensis]	2.00E-23
contig13518	1536	224	89	1.05	1.42E-09	Spore coat protein SP60 OS=Dictyostelium discoideum GN=cotC PE=1 SV=2	0.18	Collagen alpha-1(VII) chain (Fragment) OS=Clonorchis sinensis GN=CLF_109355 PE=4 SV=1	9.00E-12	collagen alpha-1(VII) chain, partial [Clonorchis sinensis]	6.00E-12
contig13459	4751	1054	421	1.04	0	Ribosomal operon-associated A protein OS=Euglena gracilis GN=roaA PE=3 SV=1	0.065	Putative von Willebrand factor type A domain-containing protein OS=Schistosoma mansoni GN=Smp_157690 PE=4 SV=1	2.00E-29	putative von Willebrand factor type A domain-containing protein [Schistosoma mansoni]	1.00E-29
contig16712	1378	75	30	1.04	0.000509013	Retrovirus-related Pol polyprotein from transposon 412 OS=Drosophila melanogaster GN=POL PE=4 SV=1	6.00E-09	Putative uncharacterized protein (Fragment) OS=Caenorhabditis brenneri GN=CAEBREN_20879 PE=4 SV=1	4.00E-12	hypothetical protein CAEBREN_20879 [Caenorhabditis brenneri]	3.00E-12
isogroup10940	1924	75	30	1.04	0.000509013	DNA polymerase OS=Zea mays PE=3 SV=1	5.00E-08	Putative uncharacterized protein OS=Tribolium castaneum GN=TcasGA2_TC006914 PE=4	1.00E-116	hypothetical protein TcasGA2_TC006914 [Tribolium castaneum]	1.00E-116
isogroup01463	1426	756	303	1.04	0	Uncharacterized protein C2orf16 OS=Homo sapiens GN=C2orf16 PE=2 SV=3	0.002	Putative uncharacterized protein (Fragment) OS=Camponotus floridanus GN=EAG_11248 PE=4 SV=1	6.00E-15	hypothetical protein EAG_11248 [Camponotus floridanus]	4.00E-15
isogroup23048	629	147	59	1.03	1.25E-06	Retrovirus-related Pol polyprotein from transposon 412 OS=Drosophila melanogaster GN=POL PE=4 SV=1	0.005	Uncharacterized protein (Fragment) OS=Nasonia vitripennis PE=4 SV=1	2.00E-06	PREDICTED: uncharacterized protein LOC101159144 [Oryzias latipes]	5.00E-05
contig13547	1882	1444	580	1.03	0	Protease inhibitors OS=Locusta migratoria PE=1 SV=2	0.22	Collagen alpha 1VII chain OS=Echinococcus granulosus GN=EgrG_000619300 PE=4 SV=1	8.00E-09	collagen alpha 1VII chain [Echinococcus granulosus]	6.00E-09
isogroup21891	687	102	41	1.03	5.55E-05	Retrovirus-related Pol polyprotein from transposon 412 OS=Drosophila melanogaster GN=POL PE=4 SV=1	0.044	Uncharacterized protein OS=Strongylocentrotus purpuratus GN=Sp-PppL_177 PE=4 SV=1	1.00E-07	Gap-Pol polyprotein [Schistosoma japonicum]	2.00E-06
isogroup10208	2318	77	31	1.03	0.000472058	Zinc finger CCHC domain-containing protein 13 OS=Homo sapiens GN=ZCCHC13 PE=2 SV=1	5.00E-05	Putative transposase OS=Anopheles gambiae PE=4 SV=1	2.00E-55	putative transposase [Anopheles gambiae]	2.00E-55
isogroup14127	1291	77	31	1.03	0.000472058	Protein phosphatase Slingshot homolog 3 OS=Mus musculus GN=Ssh3 PE=1 SV=1	2	Paired box protein and Transposase domain containing protein OS=Haemonchus contortus GN=HCOI_00676400 PE=3 SV=1	2.00E-07	PREDICTED: uncharacterized protein LOC101237877 [Hydra magnipapillata]	2.00E-07
isogroup00839	1167	896	361	1.03	0	Zinc finger CCCH domain-containing protein 13 OS=Homo sapiens GN=ZC3H13 PE=1 SV=1	3.00E-09	LWamide neuropeptides (Fragment) OS=Harpegnathos saltator GN=EAI_16679 PE=4 SV=1	8.00E-12	hypothetical protein A306_08913, partial [Columba livia]	1.00E-15
isogroup09329	3225	544	222	1.01	0	Protein FAM115 OS=Xenopus laevis GN=fam115 PE=2 SV=1	1.00E-32	Uncharacterized protein OS=Lottia gigantea GN=LOTGIDRAFT_236481 PE=4 SV=1	2.00E-60	hypothetical protein LOTGIDRAFT_236481 [Lottia gigantea]	2.00E-60
isogroup04582	1654	242	99	1.01	1.17E-09	Transcription factor 7 OS=Homo sapiens GN=TCF7 PE=1 SV=3	7.00E-35	HMG protein TCF/LEF (Fragment) OS=Dugesia japonica GN=03133HH PE=2 SV=1	1.00E-126	HMG protein TCF/LEF [Dugesia japonica]	1.00E-126
isogroup16797	1030	78	32	1.00	0.000576628	Retrovirus-related Pol polyprotein from transposon 412 OS=Drosophila melanogaster GN=POL PE=4 SV=1	2.00E-17	Putative uncharacterized protein OS=Tribolium castaneum GN=TcasGA2_TC001491 PE=4 SV=1	8.00E-24	hypothetical protein TcasGA2_TC001491 [Tribolium castaneum]	6.00E-24
