# Supplementary material for: Unusually Large Number of Mutations in Asexually Reproducing Clonal Planarian Dugesia japonica
Source: PLoS One. 2015 Nov 20;10(11):e0143525. doi: 10.1371/journal.pone.0143525 (PMC4654569; doi:10.1371/journal.pone.0143525)
Supplement: S4 Table — (PDF) [file pone.0143525.s009.pdf]

Codon table		Random SNP simulation		*D. japonica* codon usage		*D. japonica* SNP simulation	
Amino-acid (1 letter)	Codon	Count of synonymous SNPs	Count of non-synonymous SNPs	Codon frequency for each amino acid (%)	Codon frequency for all amino acids (%)	Fraction of synonymous SNPs (%)	Fraction of non-synonymous SNPs (%)
Ala (A)	GCA	3	6	38.1	1.45	0.48	0.97
	GCC	3	6	12.1	0.46	0.15	0.31
	GCG	3	6	9.4	0.36	0.12	0.24
	GCT	3	6	40.3	1.54	0.51	1.03
Cys (C)	TGC	1	8	26.3	0.60	0.07	0.54
	TGT	1	8	73.7	1.69	0.19	1.50
Asp (D)	GAC	1	8	18.6	0.99	0.11	0.88
	GAT	1	8	81.4	4.35	0.48	3.86
Glu (E)	GAA	1	8	83.1	5.37	0.60	4.77
	GAG	1	8	16.9	1.09	0.12	0.97
Phe (F)	TTC	1	8	28.3	1.38	0.15	1.23
	TTT	1	8	71.7	3.50	0.39	3.11
Gly (G)	GGA	3	6	46.6	1.91	0.64	1.27
	GGC	3	6	10.3	0.42	0.14	0.28
	GGG	3	6	8.1	0.33	0.11	0.22
	GGT	3	6	35.0	1.43	0.48	0.95
His (H)	CAC	1	8	24.3	0.54	0.06	0.48
	CAT	1	8	75.7	1.67	0.19	1.48
Ile (I)	ATA	2	7	29.9	2.58	0.57	2.00
	ATC	2	7	16.5	1.42	0.32	1.11
	ATT	2	7	53.5	4.61	1.02	3.58
Lys (K)	AAA	1	8	82.0	6.57	0.73	5.84
	AAG	1	8	18.0	1.44	0.16	1.28
Leu (L)	CTA	4	5	9.1	0.85	0.38	0.47
	CTC	3	6	6.4	0.60	0.20	0.40
	CTG	4	5	6.3	0.59	0.26	0.33
	CTT	3	6	15.7	1.46	0.49	0.97
	TTA	2	7	38.5	3.58	0.80	2.78
	TTG	2	7	24.0	2.24	0.50	1.74
Met (M)	ATG	0	9	100.0	2.20	0.00	2.20
Asn (N)	AAC	1	8	19.4	1.47	0.16	1.30
	AAT	1	8	80.6	6.08	0.68	5.40
Pro (P)	CCA	3	6	47.1	1.71	0.57	1.14
	CCC	3	6	8.1	0.29	0.10	0.20
	CCG	3	6	14.5	0.53	0.18	0.35
	CCT	3	6	30.3	1.10	0.37	0.73
Gln (Q)	CAA	1	8	77.6	3.01	0.33	2.68
	CAG	1	8	22.4	0.87	0.10	0.77
Arg (R)	AGA	2	7	43.8	1.84	0.41	1.43
	AGG	2	7	7.6	0.32	0.07	0.25
	CGA	4	5	23.3	0.98	0.43	0.54
	CGC	3	6	5.1	0.21	0.07	0.14
	CGG	4	5	5.3	0.22	0.10	0.12
	CGT	3	6	15.0	0.63	0.21	0.42
Ser (S)	AGC	1	8	6.6	0.56	0.06	0.50
	AGT	1	8	21.7	1.85	0.21	1.64
	TCA	3	6	29.5	2.52	0.84	1.68
	TCC	3	6	7.9	0.68	0.23	0.45
	TCG	3	6	9.2	0.79	0.26	0.52
	TCT	3	6	25.0	2.13	0.71	1.42
Thr (T)	ACA	3	6	42.0	2.12	0.71	1.41
	ACC	3	6	11.9	0.60	0.20	0.40
	ACG	3	6	10.1	0.51	0.17	0.34
	ACT	3	6	36.1	1.82	0.61	1.22
Val (V)	GTA	3	6	25.3	1.29	0.43	0.86
	GTC	3	6	11.8	0.60	0.20	0.40
	GTG	3	6	16.1	0.82	0.27	0.55
	GTT	3	6	46.8	2.39	0.80	1.59
Trp (W)	TGG	0	9	100.0	1.01	0.00	1.01
Tyr (Y)	TAC	1	8	22.2	0.81	0.09	0.72
	TAT	1	8	77.8	2.85	0.32	2.54
Stop (*)	TAA	2	7	52.0	0.10	0.02	0.08
	TAG	1	8	15.0	0.03	0.00	0.03
	TGA	1	8	33.1	0.07	0.01	0.06
Total frequency		24.0%	76.0%			20.3%	79.7%
