# Supplementary material for: Unusually Large Number of Mutations in Asexually Reproducing Clonal Planarian Dugesia japonica
Source: PLoS One. 2015 Nov 20;10(11):e0143525. doi: 10.1371/journal.pone.0143525 (PMC4654569; doi:10.1371/journal.pone.0143525)
Supplement: S5 Table — (PDF) [file pone.0143525.s010.pdf]

Codon position	# of SNPs		# of heterozygous SNPs		# of filtered heterozygous SNPs	
1st	84,756	25.7%	82,077	25.5%	51,691	25.5%
2nd	69,000	20.9%	66,562	20.7%	41,796	20.6%
3rd	176,623	53.5%	173,073	53.8%	108,947	53.8%
