# Supplementary material for: Unusually Large Number of Mutations in Asexually Reproducing Clonal Planarian Dugesia japonica
Source: PLoS One. 2015 Nov 20;10(11):e0143525. doi: 10.1371/journal.pone.0143525 (PMC4654569; doi:10.1371/journal.pone.0143525)
Supplement: S6 Table — (PDF) [file pone.0143525.s011.pdf]

Isogroup	Forward primer	Reverse primer
isogroup09578	GTTCTCTATCGGTTTCTGAGGAGTATTTAG	GGAGAAATATTGCATGAATTTGCC
isogroup09721	TGACCACAATGGAGCGACTTT	GTGGGACTGGTTTAGCGTTTGTA
isogroup09800	GGAGCTCGTCCGGATCTAGAA	CGAGCTGACTGTACTTGTCGGAA
isogroup10556	TCCAATTGTTCGGGACCTCC	AATTGTTTTACACAATCAACGAAATCG
isogroup18798	GCTTAACACCGACTCAAGTAGGGA	TTTTGCAGCAGCAGCTCTGTC
isogroup14546	CATTGCAGCGATACCTGACATG	TGAATTTGTCCGGAATCGGTC
isogroup17454	TTGCTCTCTTTACGCTCCGGT	CATAGTTCCCTGCATGGTGGAA
isogroup13916	TATCGGTGATGGATGATTGCC	CACCTTCGACGAATCATAGCG
isogroup09861	GTATGGATTAGCATTATTGAATTGTGCTC	AAACTCCACCGCAGTACTTTCAAC
isogroup14819	GAGTCGTAAGGCTTCTCTCCAACA	CTGAAATCGAATACTGCAACCTGA
isogroup21204	GATTTATTTGATCGCCAATGCTG	CAAAGTGATGCCCACTTCTCATC
isogroup10589	TGCTTCGGTGTTATATTTACTGTTGG	ACACAAAACGTCTTGGAGCATTCT
isogroup19020	CGGTGAAATAGCTAATGCAGCA	TTGAAACCAAACAAAAAATGGGA
isogroup05208	TTTGGCATTCTTGTCCGATTGTA	GCGAGCAGATGCGAAGATTT
contig13483	CCAAGCTGAATTTTTCTTACGGC	AAGGATGTCAATGTGTCAAACAAATATTT
contig13535	CCCATCTCGAATGAATCCTTTATTG	CTCGGCGTCCGTGTTATGAT
contig14746	GAGTCGTAAGGCTTCTCTCCAACA	CTGAAATCGAATACTGCAACCTGA
